# Improved cerebrovascular reactivity mapping using coherence weighted general linear model in the frequency domain

**DOI:** 10.1016/j.neuroimage.2023.120448

**Published:** 2023-11-10

**Authors:** Botian Xu, Chau Vu, Matthew Borzage, Clio González-Zacarías, Jian Shen, John Wood

**Affiliations:** a Department of Biomedical Engineering, University of Southern California, Los Angeles, CA, United States; b Department of Pediatrics and Radiology, Children’s Hospital Los Angeles, Los Angeles, CA, United States; c Keck School of Medicine, University of Southern California, Los Angeles, CA, United States; d Division of Neonatology, Department of Pediatrics, Fetal and Neonatal Institute, Children’s Hospital Los Angeles, Los Angeles, CA, United States; e Neuroscience Graduate Program, University of Southern California, Los Angeles, CA, United States

**Keywords:** Cerebrovascular reactivity, Carbon dioxide, Bold, General linear model, Fourier analysis, Signal coherence

## Abstract

Cerebrovascular reactivity (CVR) is a prognostic indicator of cerebrovascular health. Estimating CVR from endogenous end-tidal carbon dioxide (CO_2_) fluctuation and MRI signal recorded under resting state can be difficult due to the poor signal-to-noise ratio (SNR) of signals. Thus, we aimed to improve the method of estimating CVR from end-tidal CO_2_ and MRI signals. We proposed a coherence weighted general linear model (CW-GLM) to estimate CVR from the Fourier coefficients weighted by the signal coherence in frequency domain, which confers two advantages. First, it requires no signal alignment in time domain, which simplifies experimental methods. Second, it limits the GLM analysis within the frequency band where CO_2_ and MRI signals are highly correlated, which automatically suppresses noise and nuisance signals. We compared the performance of our method with time-domain GLM (TD-GLM) and frequency-domain GLM (FD-GLM) in both synthetic and *in-vivo* data; wherein we calculated CVR from signals recorded under both resting state and sinusoidal stimulus. In synthetic data, CW-GLM has a remarkable performance on CVR estimation from narrow band signals with a mean-absolute error of 0.7 % (gray matter) and 1.2 % (white matter), which was lower than all the other methods. Meanwhile, CW-GLM maintains a comparable performance on CVR estimation from resting signals, with a mean-absolute error of 4.1 % (gray matter) and 8 % (white matter). The superior performance was maintained across the 36 *in-vivo* measurements, with CW-GLM exhibiting limits of agreement of −16.7 % – 9.5 % between CVR calculated from the resting and sinusoidal CO_2_ paradigms which was 12 % – 209 % better than current time-domain methods. Evaluating of the cross-coherence spectrum revealed highest signal coherence within the frequency band from 0.01 Hz to 0.05 Hz, which overlaps with previously recommended frequency band (0.02 Hz to 0.04 Hz) for CVR analysis. Our data demonstrates that CW-GLM can work as a self-adaptive band-pass filter to improve CVR robustness, while also avoiding the need for signal temporal alignment.

## 1. Introduction

Cerebrovascular reactivity (CVR) measures the ability of vessels to dilate or constrict in response to a stimulus, which is an important prognostic indicator of cerebrovascular health (Liu et al., 2019). Impaired CVR reflects that the brain is vulnerable to acute interruptions in oxygen delivery, or increases in metabolic demand. Measuring the CVR could help identify patients at cerebrovascular risk for the development of stroke (Geranmayeh et al., 2015; Krainik et al., 2005) and allow escalation of care prior to irreversible brain damage in flow limiting disease processes such as carotid artery stenosis (De Vis et al., 2015; Donahue et al., 2013; Gupta et al., 2012; Mandell et al., 2008; Mikulis et al., 2005) and Moya-Moya disease (Liu et al., 2021; Noguchi et al., 2015).

CVR measurement typically requires a metered physiological challenge to increase cerebral blood flow, and a means of assessing the change in blood flow. Common physiological challenges include acetazolamide injection (Settakis et al., 2003) and carbon dioxide (CO_2_) manipulations (Liu et al., 2019). CO_2_ can be increased by asking a patient to hold their breath and elevate their endogenous CO_2_, by providing a Douglas bag filled with exogenous CO_2_, or using a purpose-built computer-controlled gas blender to deliver the CO_2_. CO_2_ inhalation has been increasingly used as the stimulus in CVR studies because it efficiently produces vasodilation and is compatible with magnetic resonance imaging (MRI). Unfortunately, the equipment of CO_2_ inhalation is cumbersome to use, uncomfortable, and unsuitable for young children. One can avoid CO_2_ inhalation by measuring endogenous fluctuations in end-tidal CO_2_ during resting breathing (Golestani et al., 2016; Liu et al., 2017, 2020; 2021; Tong et al., 2011), which is cheaper, easier for patients, and applicable to pediatrics. However, without strong stimuli, the recorded signals are usually distorted by noise and standard analysis methods work poorly. Recent work using coach-breathing maneuvers and tailored band-pass filtering has improved CVR measurements from resting state acquisitions but equal performance to metered CO_2_ challenges has not been demonstrated (Liu et al., 2020).

CVR is often calculated from the linear fit between CO_2_ stimulus and BOLD MRI signal response in the time-domain (Duffin et al., 2015; Golestani et al., 2016; Liu et al., 2017, 2020; 2021; Tong et al., 2011). This requires temporal alignment of the input and output signals, which can be difficult when signal is noisy or distorted. Several transfer function analysis-based methods (Blockley et al., 2011, 2017; Duffin et al., 2015) were proposed to produce CVR maps from the frequency domain, but they either only calculated the transfer function gain (Duffin et al., 2015) or limited CVR analysis to the fundamental frequency of an applied CO_2_ square wave or sinusoid (Blockley et al., 2011, 2013); neither method has been applied to resting state CVR measurements. A general frequency-based approach (Xu et al., 2022), which applies general linear model (GLM) in the magnitude of the signal spectrum, was proposed to avoid challenges with signal alignment and is well-suited for resting-state CVR analysis. However, the method treats all the frequencies equally, so it does not suppress noise and other unimportant signal components, which are often a considerable portion of the resting state BOLD signal.

In this paper, we proposed a new method named coherence weighted GLM (CW-GLM), which estimates CVR using signal coherence as a filter to select the frequency bands in which the CO_2_ and MRI signal are highly correlated. We postulated that this approach would improve on frequency domain GLM (FD-GLM) (Xu et al., 2022) in resting BOLD CVR measurements where the CO_2_ stimulus has a broader bandwidth.

We studied 36 subjects using a 700 second BOLD image divided into a 6.5 min resting state phase followed by a 4 min controlled sinusoidal manipulation of end-tidal CO_2_ and 70 s of recovery. This paradigm allowed us to compare the performance of multiple CVR post-processing algorithms under high and low signal-to-noise ratio (SNR) conditions. We also use these real-world data to create physiologically-grounded Monte-Carlo simulations of the CVR task for absolute quantitation of algorithm performance.

## 2. Materials and methods

### 2.1. Study population

The study was approved by the institutional review board, and all data were obtained under appropriate documentation of informed consent. Our study population were 24 volunteers who previously underwent Fontan repair for single ventricle heart disease (age = 20.6 ± 2.8, 14 males, 10 females), as well as 12 health controls (age = 21.0 ± 4.0, 7 males, 5 females) whose age, sex and ethnicity were matched to the Fontan cohort. Other demographic and laboratory data were collected, but were not pertinent to this manuscript.

### 2.2. Image acquisition and preprocessing

Brain MRI were performed on a 3T Philips Achieva D-Stream with a 32 element head coil. BOLD images were continuously acquired for 700 s using a signal echo protocol: TR/TE = 1500/30 ms, resolution = 1.9 × 1.9 × 2.5 mm, simultaneous multislice acceleration factor of 5. T1-weighted images were acquired for co-registration using the following parameters: TE = 3.8 ms, TR = 8.3 ms, resolution = 1 × 1 × 1 mm, SENSE = 2. The BOLD images were first preprocessed using FMRIB Software Library (FSL) and included (i) slice-time correction, (ii) rigid motion correction, (The motion regressors and their derivatives as nuisance variables were removed. The degree of freedom is 6.) (iii) co-registration to the corresponding T1 image, (iv) co-registration to the MNI152 template space, (v) normalization and smoothing the registered BOLD images using Analysis of Functional NeuroImages (AFNI) software. (To spatially smooth the BOLD images, a 5 × 5 Gaussian kernel was applied.)

### 2.3. End-Tidal carbon dioxide data collection

**Fig. 1.**
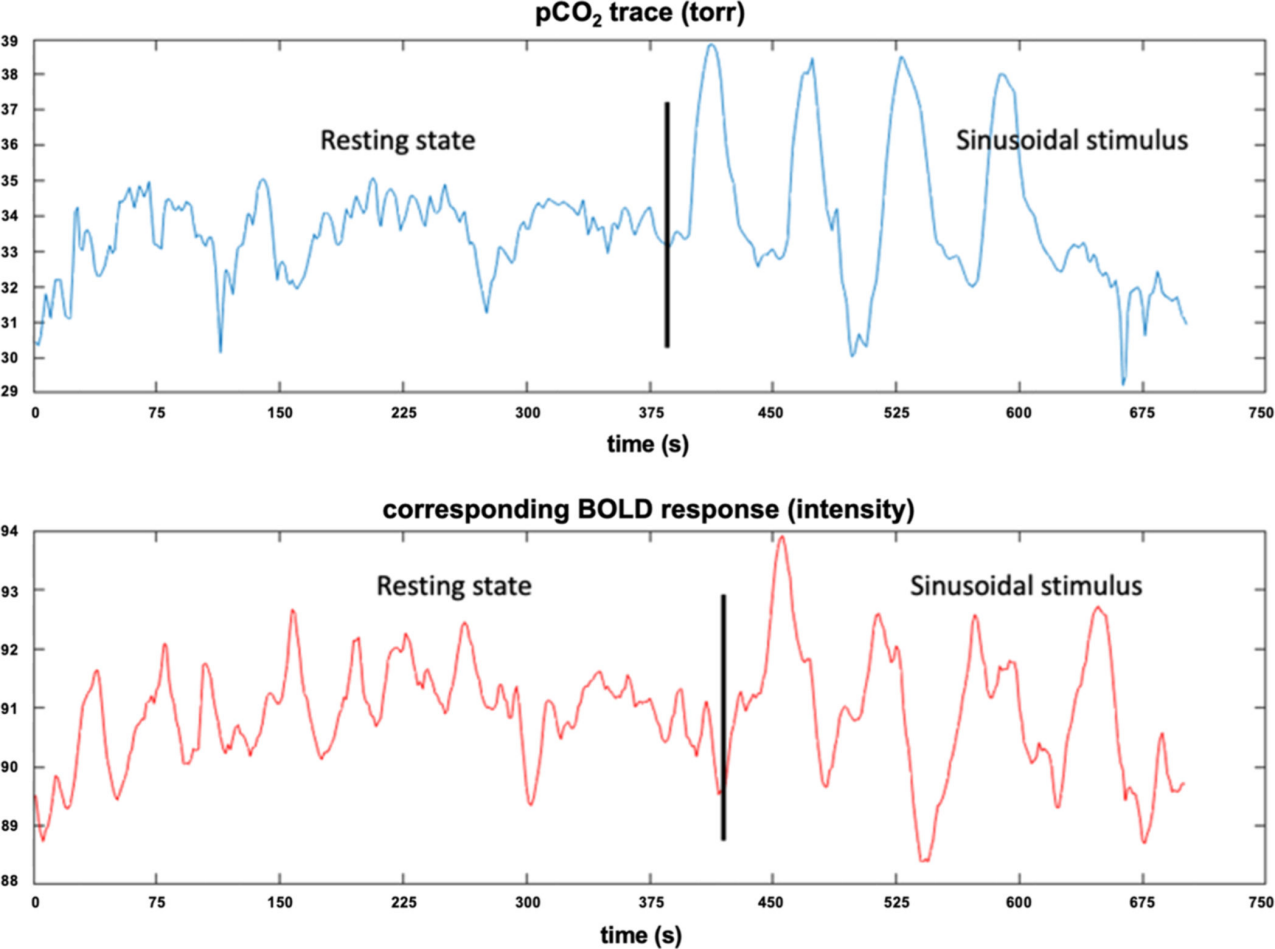
The paradigm of CVR experiment. End-tidal CO_2_ level (top) and BOLD MRI signal (bottom) are recorded under both a 6.5 min resting state and a 4-cycle sinusoidal stimulus with 1 min per cycle. The offset between the vertical black lines comes from the hemodynamic response delay and the instrumental delay; with the RespirAct, the instrumental delay is negligible compared with the HRF delay.

To validate the generalizability of CVR estimation methods, the end-tidal CO_2_ levels under both resting state and controlled CO_2_ stimuli were recorded by a computer-controlled gas blender (RespirAct, 2023). During the first portion of the resting state acquisition, the RespirAct was undergoing a preparation phase and did not control end-tidal CO_2_. After establishing the subjects “natural” end tidal CO_2_ level, the RespirAct used prospective targeting to minimize subject end tidal CO_2_ fluctuation during the remaining resting state acquisition; nominal targeting precision is ±1 torr. The exact time of transition between prep phase and targeting phase varied slightly with patient breathing pattern but was roughly 50 % of the resting sequence. During the 4 min sinusoidal CO_2_ challenge, the same device created a 4-cycle sinusoidal paradigm of CO_2_ oscillating between resting pCO_2_ and 10 torr above resting pCO_2_, followed by a period of 70 s of recovery. The CO_2_ sinusoid was biased five torr above the resting end-tidal CO_2_ to produce a consistently positive respiratory drive. The CO_2_ data were resampled and interpolated to the BOLD sampling rate (Fig. 1).

### 2.4. CVR analysis

Over short time scales, CVR is defined as the ratio between the change of BOLD MRI response and the change of end-tidal CO_2_ level (Liu et al., 2019) as shown as Eq. (1):

(1) $$CVR=\frac{△BOLD}{△EtCO_{2}}$$

where $\Delta BOLD=\frac{BOLD-BOLD_{bascline}}{BOLD_{baseline}}\times 100\%$ represents the percentage changes of BOLD signal from the baseline, and $\Delta EtCO_{2}$ represents the end-tidal CO_2_ difference between baseline and hypercapnic measurements. For the present study, the mean BOLD signal during the resting state was using for normalization $\left(BOLD_{baseline}\right)$ for both challenges.

For a large sample size, CVR can be estimated by using time domain general linear model (TD-GLM), which calculates the least-squares linear fit between temporally-aligned BOLD and CO_2_ signals. Time delays between the BOLD and CO_2_ signals (Thomas et al., 2014; Yezhuvath et al., 2009) result from instrumentation delays (e.g. in the CO_2_ sampling line), CO_2_ transit time from lung to brain, and the brain’s hemodynamic response. Therefore, careful signal alignment is essential before applying a voxel-wise linear fit. Typically, cross-correlation analysis is used, either globally, or voxel-by-voxel to account for delay differences among tissues. This can also be performed by an advanced software package, Rapidtide (Frederick, 2016; Tong et al., 2011).

Alignment challenges can be overcome by formulating the problem in the frequency domain. That is, let x(t) be the end-tidal CO_2_ change, y(t) be the corresponding change of BOLD signal during the time, and τ be the voxel wise delay between the BOLD and CO_2_ signals. The linear relationship in time domain (which refers to TD-GLM) can be represented as:

(2) y(t)=CVR⋅x(t−τ)

Applying the Fourier transform on both sides of the equation, the CO_2_ and BOLD signals can be separated into the magnitude portion and phase components:

(3) $$Y(\omega )=CVR\cdot X(\omega )e^{-j\omega \tau }$$

In frequency space, CVR is a scalar relationship between the input and output spectra, independent of phase. In contrast, signal delays (by instrumentation or hemodynamic response) are reflected by the slope, t, of the resulting linear phase shift. If we only consider the magnitude of Eq. (3) and apply a GLM in frequency domain (FD-GLM) we obtain:

(4) |Y(ω)|=CVR⋅|X(ω)|+c+ε

where |Y(ω)| and |X(ω)| are the magnitude spectra, c is the estimated intercept, and ε is the estimated error arising from imperfect measurements of the CO_2_ signal and fluctuations in the BOLD signal independent of CO_2_ change. Epsilon is modelled as a white Gaussian noise process, and c is equal to 0 when $x(t)=\Delta EtCO_{2}$ and y(t)=ΔBOLD. Thus, CVR can be estimated solely by |Y(ω)| and |X(ω)| using the least-squares linear fit, eliminating the need for signal time shifting. To stabilize GLM in the frequency domain, it is critically important to include the DC Fourier component in the least-squares linear fit.

Note that Eq. (4) weights the contributions of each frequency equally. However, blood vessel relaxation in response to CO_2_ change is constrained to a relatively narrow frequency range that can be identified through the signal coherence between the BOLD and CO_2_ signals. Some of the BOLD fluctuations result from spontaneous fluctuations in neural activity (Liu et al., 2019), rather than changes in the end-tidal CO_2_ and these represent physiological noise with respect to CVR calculation. Liu and colleagues have empirically identified 0.02 – 0.04 Hz as being “enriched” for CVR information (Liu et al., 2017). However, the exact range is likely subject dependent. To remove noise and unimportant signals on a subject specific basis, we introduce a coherence weighted GLM (CW-GLM) shown as Eq. (5):

(5) $$\frac{C_{xy}(\omega )}{\int C_{xy}(\omega )d\omega }\cdot |Y(\omega )|=CVR\cdot |X(\omega )|+c+\varepsilon$$

(6) $$C_{xy}(\omega )=\frac{{\left|G_{xy}(\omega )\right|}^{2}}{G_{xx}(\omega )G_{yy}(\omega )}$$

where $C_{xy}(\omega )$ is the signal coherence shown in Eq. (6). $C_{xy}(\omega )$ is the cross-spectral density, $G_{xx}(\omega )$ and $G_{yy}(\omega )$ are the respective auto-spectral densities. Note that although individual values of $C_{xy}(\omega )$ are between 0 and 1, their sum might not equal to 1. Therefore, the _∫_ coherence must be normalized by $\int C_{xy}(\omega )d\omega$. Essentially, we have created a matched-filter design in the frequency domain to optimally emphasize the spectral bands important to CVR against background physiological noise.

Although Eq. (2) is the most commonly used mathematical representation of the brain BOLD signal in a CVR experiment, it is a simplified model that does not consider cerebrovascular hemodynamics. A more general case can be written by describing the brain as a linear system with an impulse response function, h(t) as follows:


(7) y(t)=h(t)*x(t)

where h(t) represents the brain’s response to the changes in P_a_CO_2_ (approximated by end-tidal CO_2_). Transforming this relationship to the frequency domain yields

(8) Y(ω)=H(ω)X(ω)

where H(ω) is the hemodynamic “filter” response produced by brain vasculature. By inspection, one can see that Eqs. (2) and (3) are equivalent to Eqs. (7) and (8) if h(t)=CVR⋅δ(t−τ) and $H(\omega )=CVR\cdot e^{-j\omega \tau }$. This represents an all-pass filter with linear phase response proportional to the delay. However, studies using step changes in CO_2_ (Duffin et al., 2015) have clearly demonstrated that the brain’s response to CO_2_ can be effectively modeled by a single-pole low-pass filter as follows:

(9) $$h(t)=CVR\cdot e^{-\alpha (t-\tau )}u(t-\tau )$$

where α is a first order low pass response parameter. In the frequency domain this yields

(10) $$H(\omega )=CVR\cdot e^{-j\omega \tau }\cdot \frac{\alpha }{\alpha +j\omega }$$

**Fig. 2.**
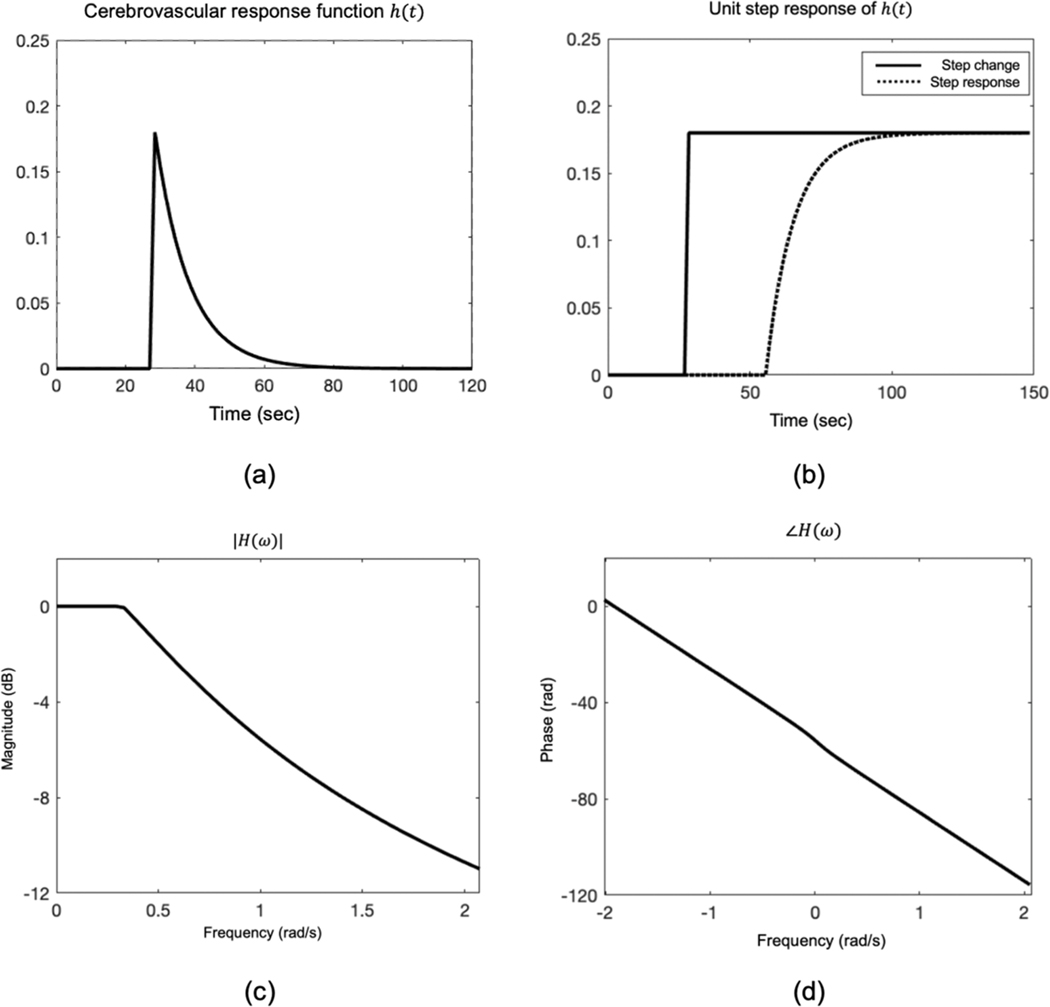
The profile of the simulated cerebrovascular response function h(t): (a) the time domain representation of h(t); (b) the unit step response of h(t) (dotted curve) and the ideal step change without h(t) (solid curve); (c) the magnitude of H(ω); (d) the phase of H(ω).

Fig. 2 demonstrates h(t), its step response, as well as its magnitude and phase characteristics. The step response to CO_2_ is intuitive, with maximum vasodilation (determined by CVR) achieved over a characteristic rise time (determined by α). The frequency response exhibits weak attenuation outside of the passband with nearly-linear phase delays. Since |H(ω)| is not all-pass, it introduces a frequency-dependent scaling factor that must be taken into account when one recasts FD-GLM and CW-GLM as follows:

(11) |Y(ω)|=|H(ω)‖X(ω)|+c+ε

(12) $$\frac{C_{xy}(\omega )}{\int C_{xy}(\omega )d\omega }\cdot |Y(\omega )|=|H(\omega )||X(\omega )|+c+\varepsilon$$

Plugging Eq. (10) into (11) and (12) yields the final form of FD-GLM and CW-GLM in frequency space

(13) $$|Y(\omega )|=CVR\cdot \left|\frac{\alpha }{\alpha +j\omega }\right|\cdot |X(\omega )|+c+\varepsilon$$

(14) $$\frac{C_{xy}(\omega )}{\int C_{xy}(\omega )d\omega }\cdot |Y(\omega )|=CVR\cdot \left|\frac{\alpha }{\alpha +j\omega }\right|\cdot |X(\omega )|+c+\varepsilon$$

In the general case, the α coefficient is unknown and would have to be estimated from the data in order to calculate FD-GLM and CW-GLM. However, since the filter response is low-order, we chose to model α as a population based constant value, positing that small intrasubject uncertainties in the filter cutoff (see Fig. 2c) would have little impact on the final CVR calculation.

Since the time and frequency domain are duals of each other, according to Eqs. (12) and (14), the normalized coherence weights can be treated as a band-pass filter applied on BOLD signal, while the hemodynamic response function (HRF) works as a low-pass filter on CO_2_ signal. Therefore, the time domain representation of the CW-GLM can be written as following equation:

(15) $$y(t)*cw(t)=CVR\cdot (x(t)*\tilde{h}(t))+p+\varepsilon$$

where cw(t) is the impulse response of the coherence function, $\tilde{h}\left(t\right)$ is the HRF excluded the CVR magnitude, and p is other physiological terms. To simplify the equation, Eq. (15) can be rewritten as:

(16) $$\tilde{y}(t)=CVR\cdot \tilde{x}(t)+p+\varepsilon$$

where $\tilde{y}\left(t\right)=y\left(t\right)*cw\left(t\right)$, and $\tilde{x}\left(t\right)=x\left(t\right)*\tilde{h}\left(t\right)$ . In this way, the CW-GLM approach can be alternatively performed in the time domain. Note that although the coherence filter and HRF will improve the signals affected by the physiologic noise, instrumental delay must be still be corrected separately from the delay produced by the HRF.

### 2.5. Comparison with time domain and frequency domain GLM methods

BOLD data were preprocessed identically for both the time and frequency domain analyses. In our TD-GLM experiment, we performed cross correlation twice for each subject. We first aligned the end-tidal CO_2_ and global BOLD signal to provide a robust correction of the dominant time delay, then we implement a constrained cross correlation to temporally align individual voxels. The median delay in gray matter was 15 s, but the time-constant was distributed log-normally, and white matter exhibited a significantly slower response. A study of the age effects on CVR delay (Thomas et al., 2014) indicates that the delay of WM to EtCO_2_ is roughly round 34.4 +/− 11.2 s in a young population. In the present study, we observed some voxels (less than 5 %) have delay up to 56 s, consistent with the Thomas data. After correction of the global shift, which removes most of the EtCO_2_ to GM delay, the purpose of the staged voxelwise alignment is to correct regional variation in GM delay as well as the larger GM-WM delay. Thomas reported GM-WM delay to be 19.1 +/− 11.6 s (upper bound 41.3 s). To be conservative, the constraint of time shifting was set less than 60 s (one period). CVR was subsequently calculated on a voxelwise basis using Eq. (1) and the delay was calculated from the sum of the linear shifts between the global and voxelwise cross-correlations. We also repeated the above analysis after pre-filtering the BOLD data between 0.02 – 0.04 Hz as suggested by Liu and colleagues (Liu et al., 2017). The band-pass filter used in the experiment was a fourth-order Butterworth filter performed in a forward-backward paradigm to achieve zero-phase filtering (filtfilt command, MATLAB).

To perform the frequency domain GLM, the BOLD data was Fourier transformed in the temporal dimension for every voxel and linearly fit to the EtCO_2_ spectrum using Eq. (11). To suppress physiological noise, we also repeated the analysis after applying the same 0.02 – 0.04 Hz band-pass filter used for the time-domain analysis.

In the frequency domain, delay maps must be calculated from the phase. In FD-GLM, the time-delay at each frequency is approximated by the unwrapped phase divided by angular frequency; the average time-delay across frequency is used to form the delay map. This process is known as the group delay and is a well-established technique in signal processing (Papoulis, 1977). When apriori information is known about the source signal, delay estimates can be improved by limiting the range of frequencies that group delay is calculated (Meloni et al., 2014). For the band-pass filtered FD-GLM, time-delay averaging is confined to the narrow passband previously associated with good CVR measurements, making it more robust.

The calculation of CW-GLM is completely analogous to the band-pass filtered FD-GLM, except the bandpass filter is replaced by the coherence function for the calculation of both CVR and delay.

In order to compare CVR estimates from the time and frequency domains, it is critical to correct for the frequency dependent scaling, $\left|\frac{\alpha }{\alpha +j\omega }\right|$ in Eqs. (13) and (14), which means that CVR calculated by FD-GLM and CW-GLM results need to be scaled by the magnitude $\left|\frac{\alpha }{\alpha +j\omega }\right|$ when comparing to TD-GLM results. While α is patient and tissue specific, we chose to evaluate method performance using a regional fixed α value, which is 0.3 *s*^− 1^ in gray matter and 0.12 s^− 1^ in white matter based on the range of values estimated from the sinusoidal stimuli. More details of α determination are discussed in Section 2.7.

This left a total of four methods to compare with the performance of CW-GLM. Details of this comparison are described in the statistics section (2.8).

### 2.6. Internal validation

Our fundamental premise was that the CVR measured during the resting state should be equal to the CVR estimated during the sinusoidal portion. The sinusoidal CO_2_ stimulus was powerful and confined to a single frequency band providing excellent CVR and delay maps. We chose to use the sinusoidally-derived values as gold standard measurements, with differences between the derived CVR and delay measurements used as markers of methodological robustness for the different methods. We posited that the best analysis methods would yield the highest correlation and lowest bias, variance, and mean absolute error for CVR and delay calculated between the resting and sinusoidal portions. To fairly compare the methods, we analyzed the resting and stimulated BOLD images independently and did not allow signal alignment from the sinusoidal fluctuations to “inform” alignment during the resting state portion.

### 2.7. Monte carlo validation

Since all real-world acquisitions contain imperfections, we used estimates of CVR and physiological noise power from this study to create a Monte-Carlo simulation framework that would allow absolute quantification of CVR estimation error. To create synthetic BOLD signals with known CVR and plausible temporal response parameters (Duffin et al., 2015), we modeled the cerebrovascular response function according to Eq. (9). We assumed that CVR was sampled from a Gaussian distribution N (0.18, 0.04)%ΔBOLD/torr CO_2_, having the same mean and standard deviation as derived from our real-world measurements from 12 health controls. The signal response to step changes in CO_2_ (Duffin et al., 2015) has been described as an exponential having a rise time of 15 s, corresponding to a low pass filter cutoff value, α, of 0.15 s^− 1^.To accommodate all conceivable variations of this value with age and disease in our simulations, we subsequently drew this value from a logarithmic-normal distribution, N(0.15, 0.1) s^− 1^, corresponding to rise times of 6 – 45 s. Since we assumed the instrumentation delay was nearly zero for the RespirAct, the linear delay term, τ, in Eq. (9) was removed.

**Fig. 3.**
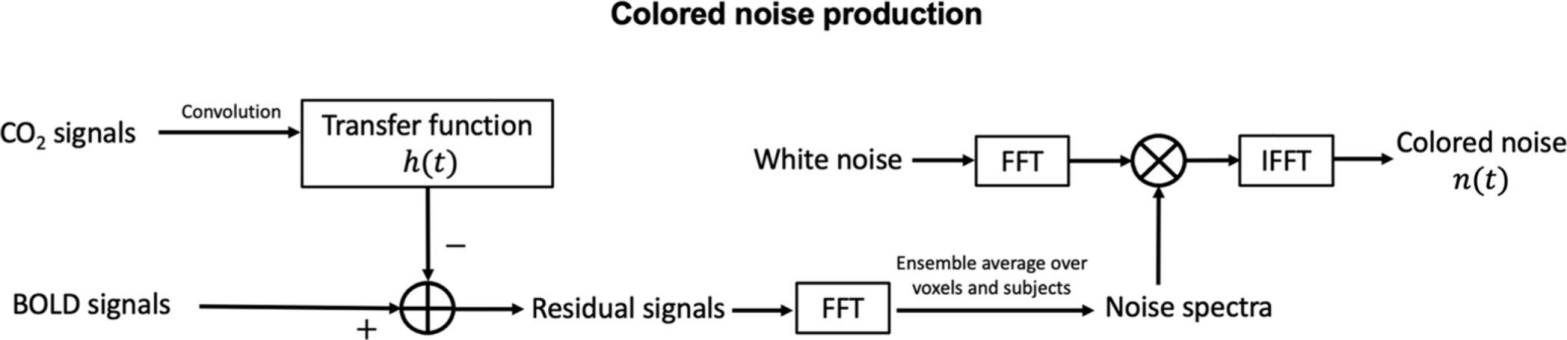
The process of physiological noise generation in Monte-Carlo simulation.

Thus, the synthetic BOLD was constructed from the convolution of cerebrovascular response function h(t) and EtCO_2_ signals corrupted by physiological noise, n(t):

(15) $$BOLD\left(t\right)=h\left(t\right)*EtCO_{2}\left(t\right)+n\left(t\right)$$

where the EtCO_2_ consisted of either a 4-min perfect sinusoidal CO_2_ signal fluctuating from 35 torr to 45 torr over a one-minute period, or a 6.5 min simulated resting CO_2_ trace. We simulated the resting CO_2_ trace as a colored noise sequence having random phase and a magnitude spectrum matched in shape and power to an ensemble average of our real-world resting state EtCO_2_ power spectra. The physiological noise in the BOLD signal, n(t), was generated by the process shown in Fig. 3. We estimated the spectral shape and power of n(t) by forming the residual of the sinusoidal BOLD signals and their corresponding, filtered (using Eq. (9)) CO_2_ signals in the time domain, transforming to the frequency domain, and calculating the ensemble average of the magnitude spectra across subjects and across voxels. This allowed to estimate the spectral envelope and SNR of the gray and white matter separately. The resultant magnitude spectrum, with zero phase, was used to filter random Gaussian white noise sequences to produce n(t). Gray matter exhibited a higher SNR (4.3 dB) but the noise spectrum was low pass, heavily overlapping the EtCO_2_ spectrum. In contrast, the SNR in the white matter was quite poor (− 1.3 dB), but was nearly white.

**Fig. 4.**
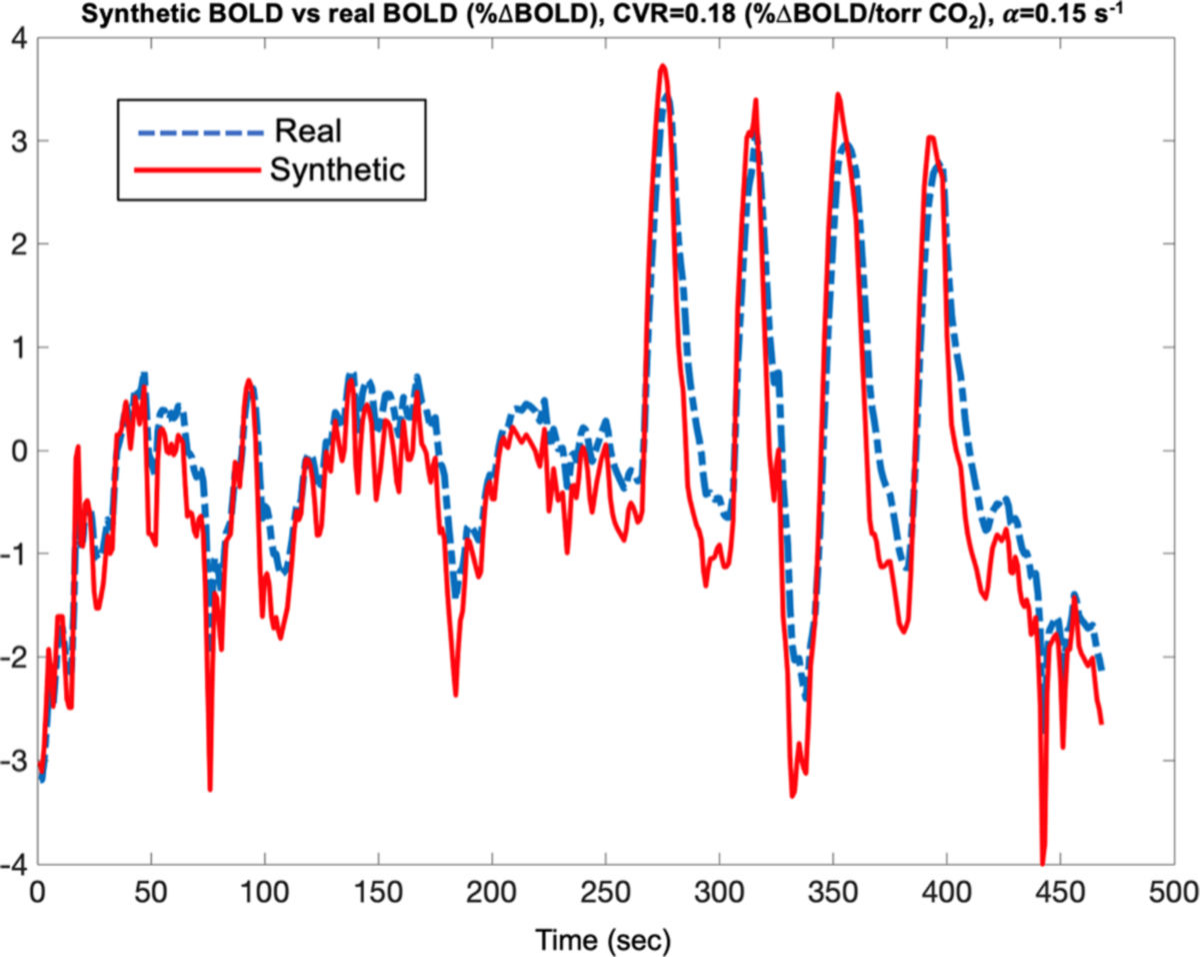
Demonstrates a real BOLD signal (dashed curve) and the Monte Carlo model with CVR and α matched to minimize mean square error over the sinusoidal portion. The overall profile of synthetic signal is plausible in both the sinusoidal and resting portions of the stimulus. The signals are normalized to percentage change from the baseline.

This framework allowed us to compare all methods in unlimited numbers of realistic BOLD signals having ground truth CVR values. 10,000 iterations were performed for each modality. Fig. 4 shows an example of the real BOLD signal (dashed curve) acquired from MRI and a BOLD signal (solid curve) synthesized from the actual CO_2_ data, where CVR, α, and τ were tuned to minimize the mean squared error in the sinusoidal portion. The differences in the red and blue curves served as the substrate for calculation of n(t).

### 2.8. Statistical analysis

For the simulated BOLD signals, the bias and mean absolute error between estimated CVR and ground truth was calculated separately for the resting and sinusoidal portion. This allowed absolute error quantification across methods. A total of seven methods were compared, the original TD-GLM, TD-GLM with band-pass filtering (TD-GLM-BP), TD-GLM with hemodynamic response function (TD-GLM-HRF), TD-GLM with band-pass filtering and HRF (TD-GLM-BP-HRF), FD-GLM, FD-GLM with band-pass filtering (FD-BP-GLM) and CW-GLM. Given the large “N” of Monte Carlo simulations, we report the effect size of the bias as a one-sample T statistic (we are comparing the bias to zero) and report the relative variability using a variance ratio statistic $\left(\text{F}=\sigma_{1}^{2}/\sigma_{2}^{2}\right)$, with $\sigma_{2}$ representing the lowest standard deviation across the methods.

The comparison of real world CVR values measured during resting state and sinusoidal CO_2_ challenge were compared to one another using Bland Altman analysis (Bland and Altman, 1986) for each CVR method; the method with the lowest variance (by two sample variance test) was judged superior. We also repeated the comparison using CVR calculated by CW-GLM as the best *in-vivo* approximation to “ground truth”. T-test was used to probe for method bias, while variance test was used to compare method accuracy across techniques.

To include the spatial information of the data and provide a more intuitive performance metric, we performed a voxel-wise correlation analysis between the resting and sinusoidal CVR and delay maps, generating both slope and r^2^ maps.

## 3. Results

### 3.1. CVR validation in synthetic data

Table 1 summarizes the Monte Carlo simulation results. The mean bias, standard deviations, and mean absolute errors of seven CVR estimation methods with respect to ground truth are shown for resting state and sinusoidal stimuli in gray matter and white matter region, respectively. Some global trends are readily apparent and intuitive. Resting CVR exhibited higher variance that sinusoidal CVR, and white matter CVR yielded poorer performance than gray matter. The largest biases are observed in the TD-GLM methods without HRF correction, underestimating the CVR value by around 30 %. HRF correction markedly improved TD-GLM in both the resting state and the sinusoidal stimuli.

With respect to variability, the frequency-based methods out-performed the time-domain methods for the sinusoidal signal, which is intuitive given the narrow-band nature of the challenge. Much less separation was observed in the resting state, with near equivalence of the TD-GLM-HRF and CW-GLM methods. Surprisedly, application of band-pass filtering worsened performance of both the time and frequency-based methods.

### 3.2. CVR validation in vivo, global CVR

**Fig. 5.**
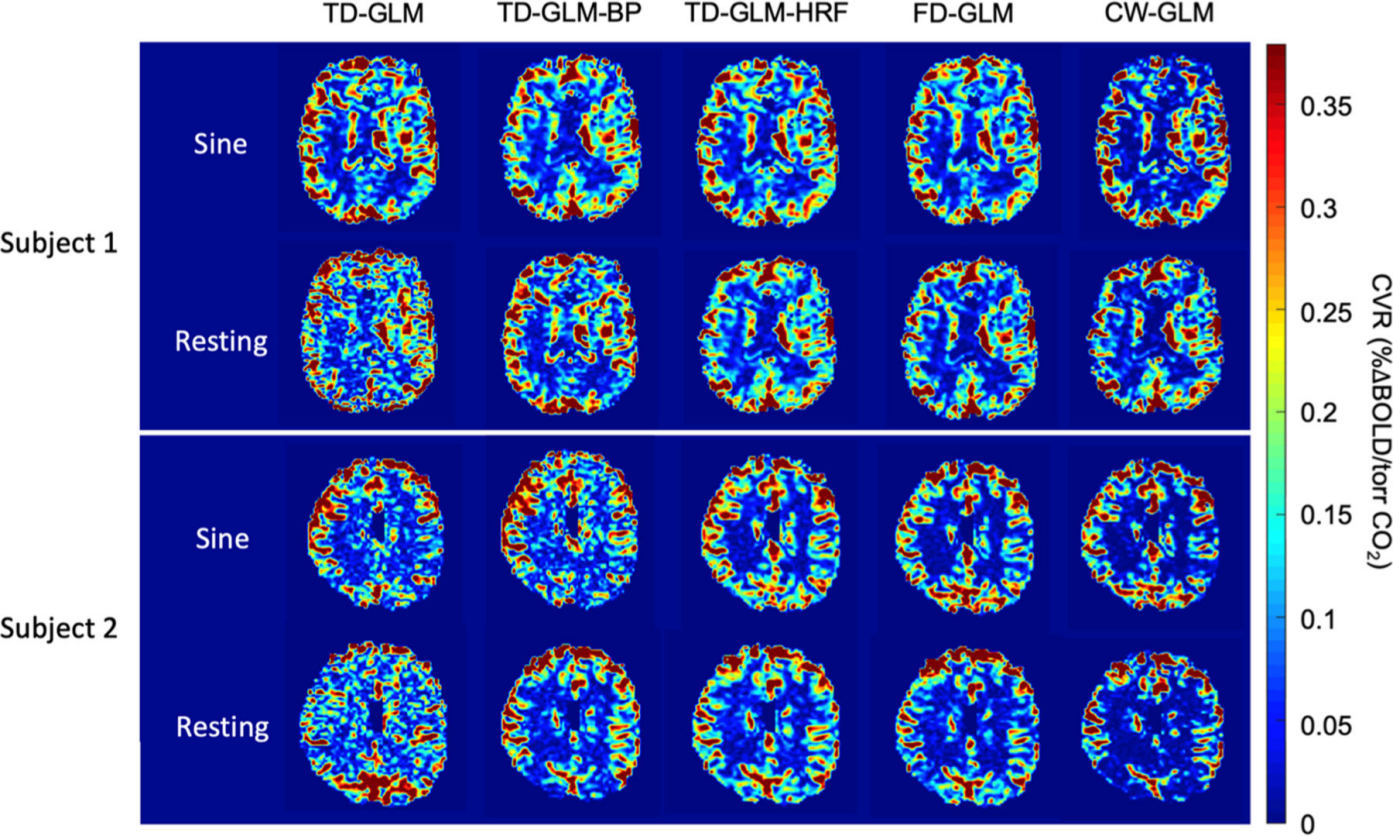
The representative slices of the CVR maps generated from different methods under a sinusoidal stimulus (top) and a resting state (bottom), respectively. (BP: with band-pass filter; HRF: with hemodynamic response function.).

To validate the *in-vivo* data, we compared resting CVR values to those obtained during the sine wave stimulus. We initially ran all analyses on healthy controls and Fontan repair subjects separately, and found there was no significant difference between the results, so the two cohorts were pooled. Due to the negative interaction of the bandpass filter on TD-GLM-HRF and FD-GLM, we chose to only present TD-GLM-BP in the main figures, along with TD-GLM, TD-GLM-HRF, FD-GLM, and CW-GLM. TD-GLM-BP-HRF and FD-GLM-BP image examples are shown in Supplementary Data (Additional CVR maps section). Fig. 5 shows several examples of different subjects’ CVR maps generated from the five selected methods. All five techniques provide robust CVR maps for the sinusoidal data. Under resting state conditions, simple TD-GLM without filtering is much noisier than the other methods, especially in the white matter. Band-pass filtering visually improves TD-GLM appearance, however CVR maps are discernably different than corresponding maps from the sinusoidal stimulus. For example, the cortical ribbon appears thicker and the white matter CVR exhibits more contrast for the sinusoid compared with resting state. HRF filtering markedly improves TD-GLM performance for the resting state data, comparable to the Monte Carlo findings.

In contrast, all the results from the frequency-based methods (FD-GLM and CW-GLM) are highly consistent between resting state and stimulus, except for slightly greater contrast observed in white matter. All frequency-domain techniques also show high similarity among themselves.

**Fig. 6.**
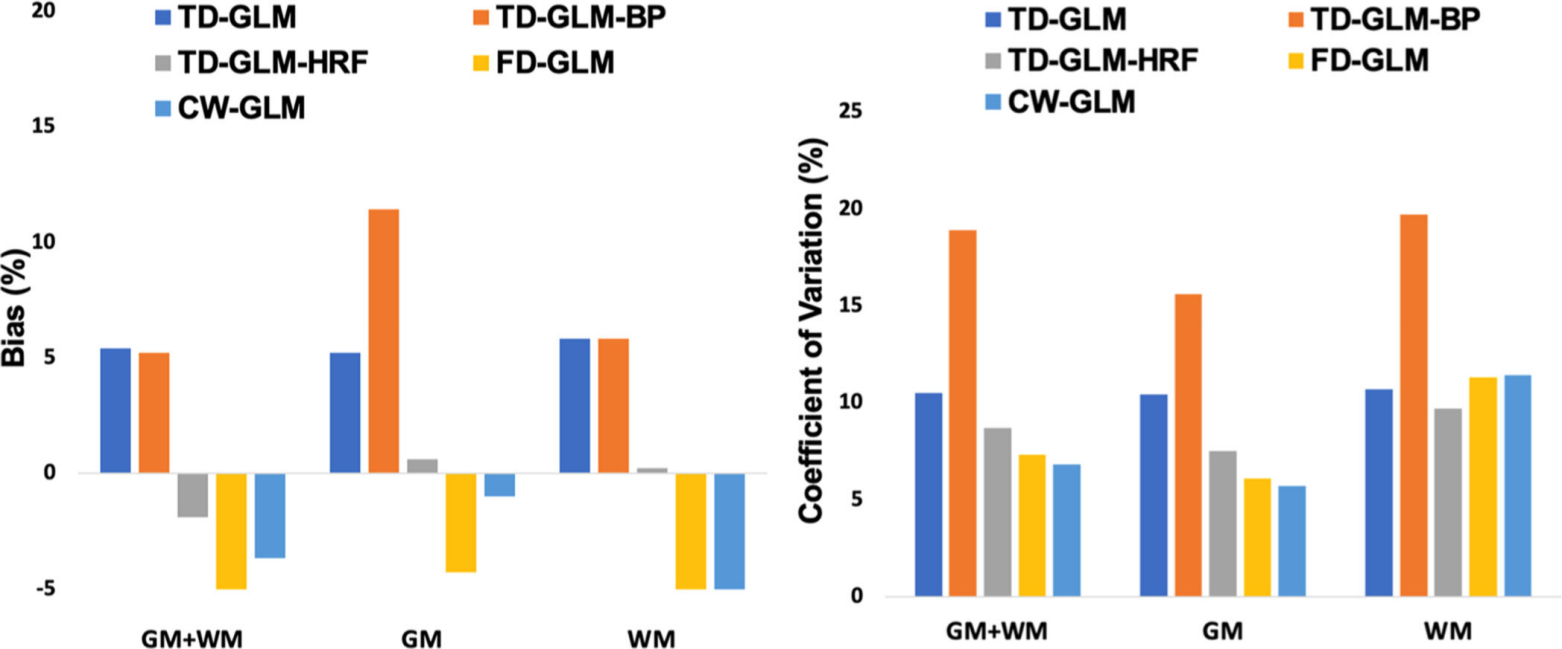
Bias and coefficient of variation between CVR values calculated from resting state and sinusoidal stimuli, reported for gray matter, white matter, and combined regions of interest.

Using the sinusoidal stimulus as a “gold standard” for each technique (comparing like versus like), we performed Bland Altman analysis between CVR values calculated during the resting state and sinusoidal CO_2_ challenges (Fig. 6). Only the TD-GLM-HRF was unbiased; the other four techniques had biases between two and six percent. Note that since we were comparing like-versus-like, the intrinsic bias from lack of HRF correction was not observed for the TD methods. The coefficient of variation differed considerably across technique (Fig. 6), with CW-GLM < FD-GLM < TD-GLM-HRF < TD-GLM < TD-GLM-BP. This relative performance among the techniques mirrored results of the Monte Carlo simulations (Table 1), with the exception that TD-GLM-HRF did not perform quite as well as the frequency domain methods. Table 2(a) contains the complete Bland Altman comparison.

### 3.3. CVR validation in vivo, regional CVR

**Fig. 7.**
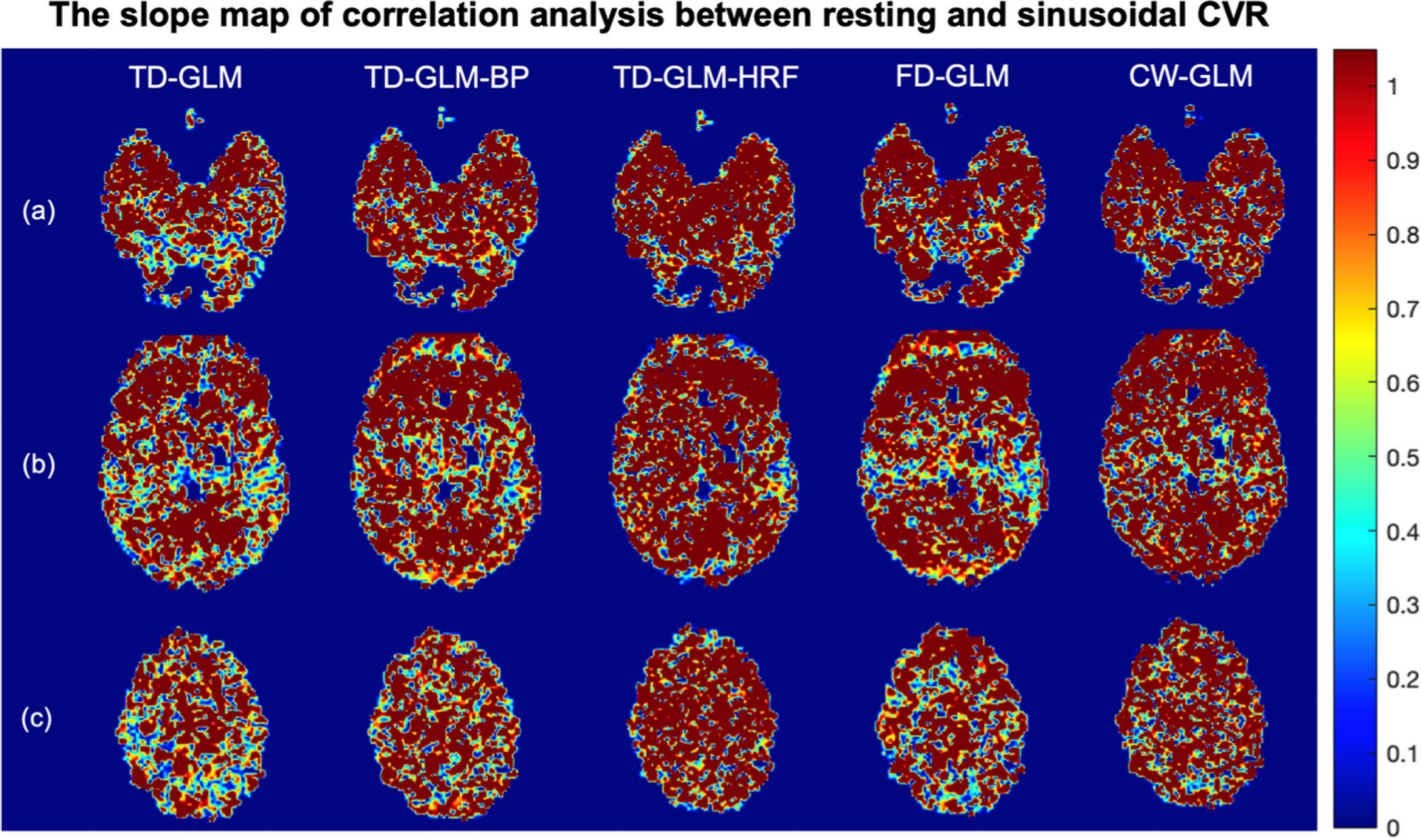
The representative slices of the slope maps generated from correlation analysis between resting and sinusoidal CVR from 36 subjects. Rows (a)(b)(c) show different slices, and different methods are indicated in each column. (BP: with band-pass filter; HRF: with hemodynamic response function.).

**Fig. 8.**
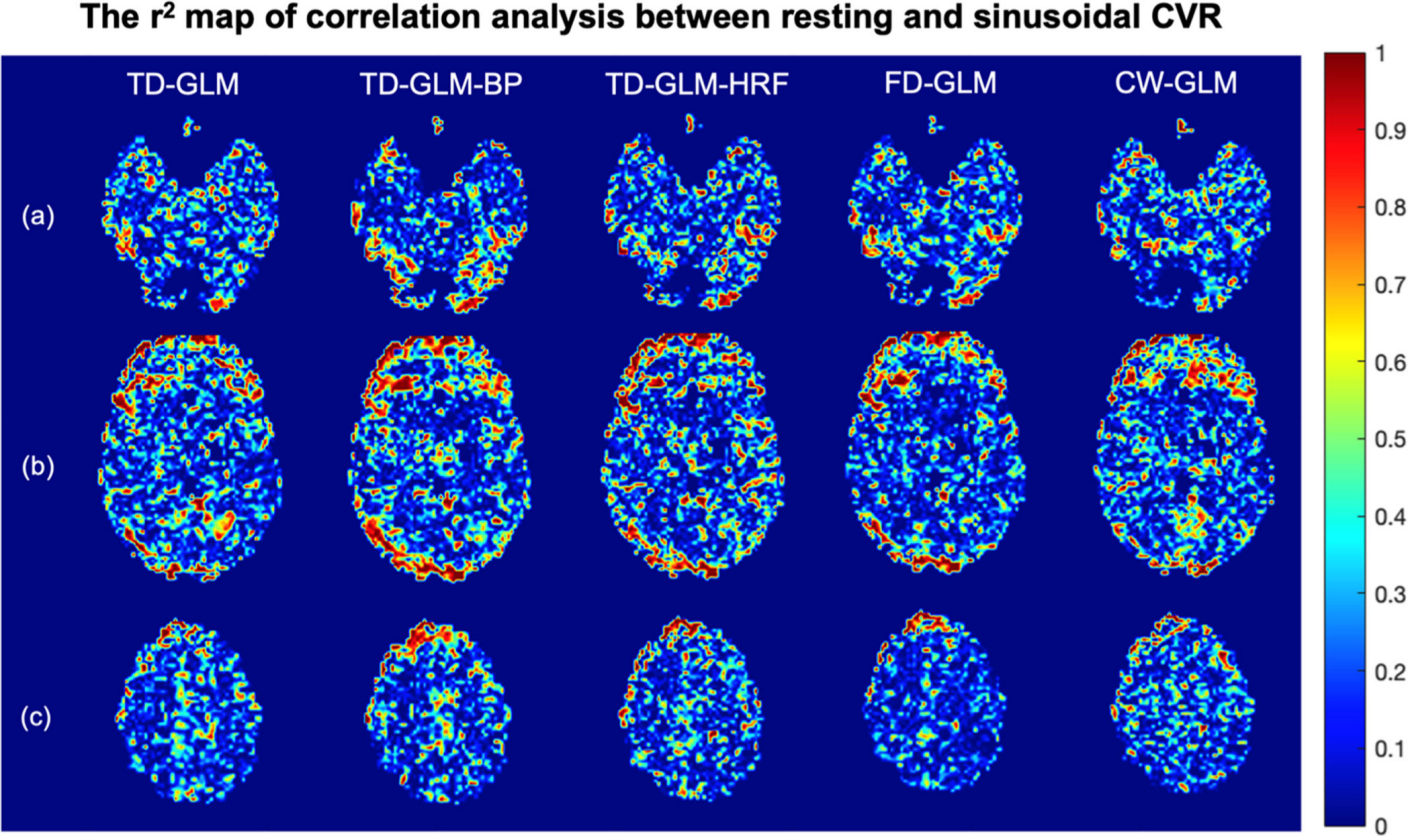
The representative slices of the ${\text{r}}^{2}$ maps generated from correlation analysis between resting and sinusoidal CVR from 36 subjects. Rows (a)(b)(c) show different slices, and different methods are indicated in each column. r>0.33 $\left(r^{2}>0.109\right)$ would be considered statistically significant. (BP: with band-pass filter; HRF: with hemodynamic response function.).

To gain more insight into regional agreement, Figs. 7 and 8 demonstrated slope and r^2^ maps of voxelwise correlation analysis between resting and sinusoidal CVR maps. In all the representations, slope values are closer to unity and r^2^ values closer to one in the gray matter than in the white matter. Table 3 summarizes the mean and standard deviation of all the voxel-wise slope and r^2^ values in gray matter and white matter, respectively. The same performance trends observed in the Monte Carlo simulations (Table 1) and Bland Altman analyses (Fig. 6, Table 2) are mirrored in Table 3.

### 3.4. CVR validation in vivo, regional delay

**Fig. 9.**
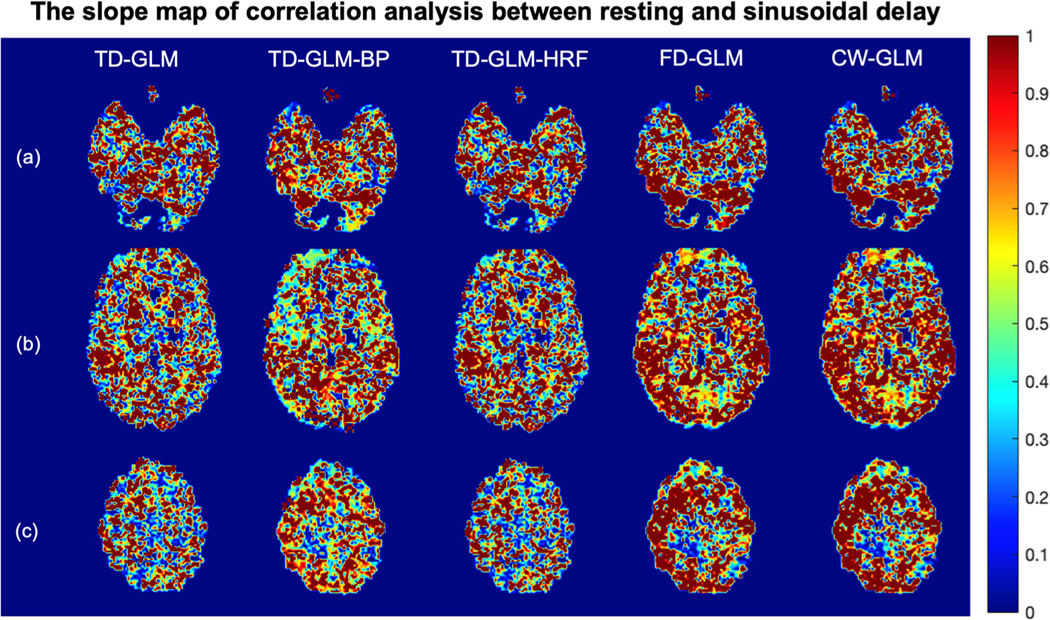
The representative slices of the slope maps generated from correlation analysis between resting and sinusoidal delay from 36 subjects. Rows (a)(b)(c) show different slices, and different methods are indicated in each column. (BP: with band-pass filter; HRF: with hemodynamic response function.).

**Fig. 10.**
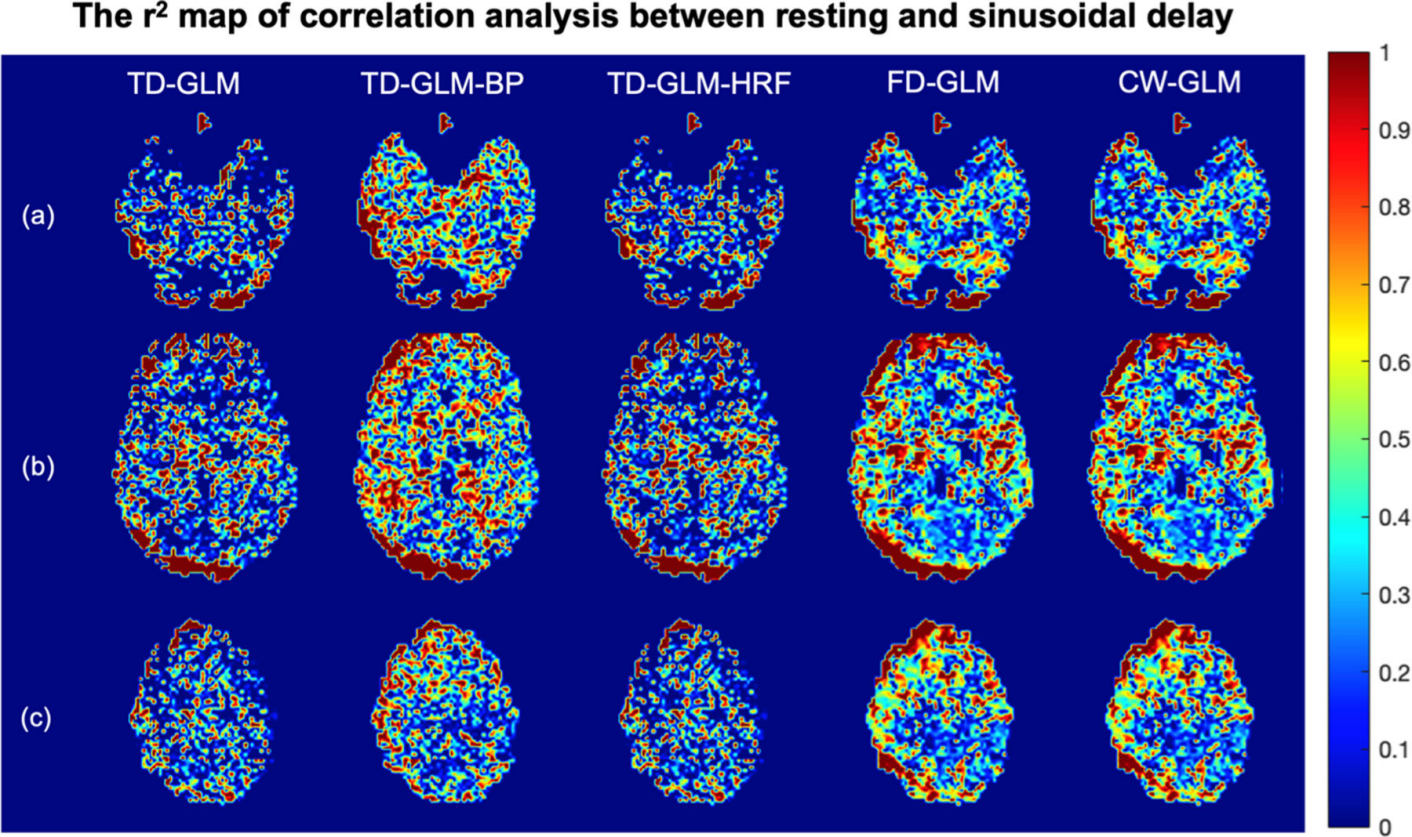
The representative slices of the ${\text{r}}^{2}$ maps generated from correlation analysis between resting and sinusoidal delay from 36 subjects. Rows (a)(b)(c) show different slices, and different methods are indicated in each column. $r>0.33\;\left({\text{r}}^{2}>0.109\right)$ would be considered statistically significant. (BP: with band-pass filter; HRF: with hemodynamic response function.).

Similar to the comparison of the CVR magnitude maps, the slope (Fig. 9) and r^2^ (Fig. 10) of correlation analysis between resting and sinusoidal delay are shown. Many of the slope values are unity, indicating that the delay estimates are mostly unbiased. However, the r^2^ maps suggest that time delay estimation from resting state data is not as robust as for CVR, using either time domain or frequency domain approaches, particularly in the white matter. Table 4 summarizes the mean and standard deviation of all the voxel-wise slope and r^2^ values. The overall performance profile of the five methods mirrors the CVR analyses, with top performance found in CW-GLM (r^2^ = 0.60 – 0.76).

### 3.5. CVR comparisons across techniques

**Fig. 11.**
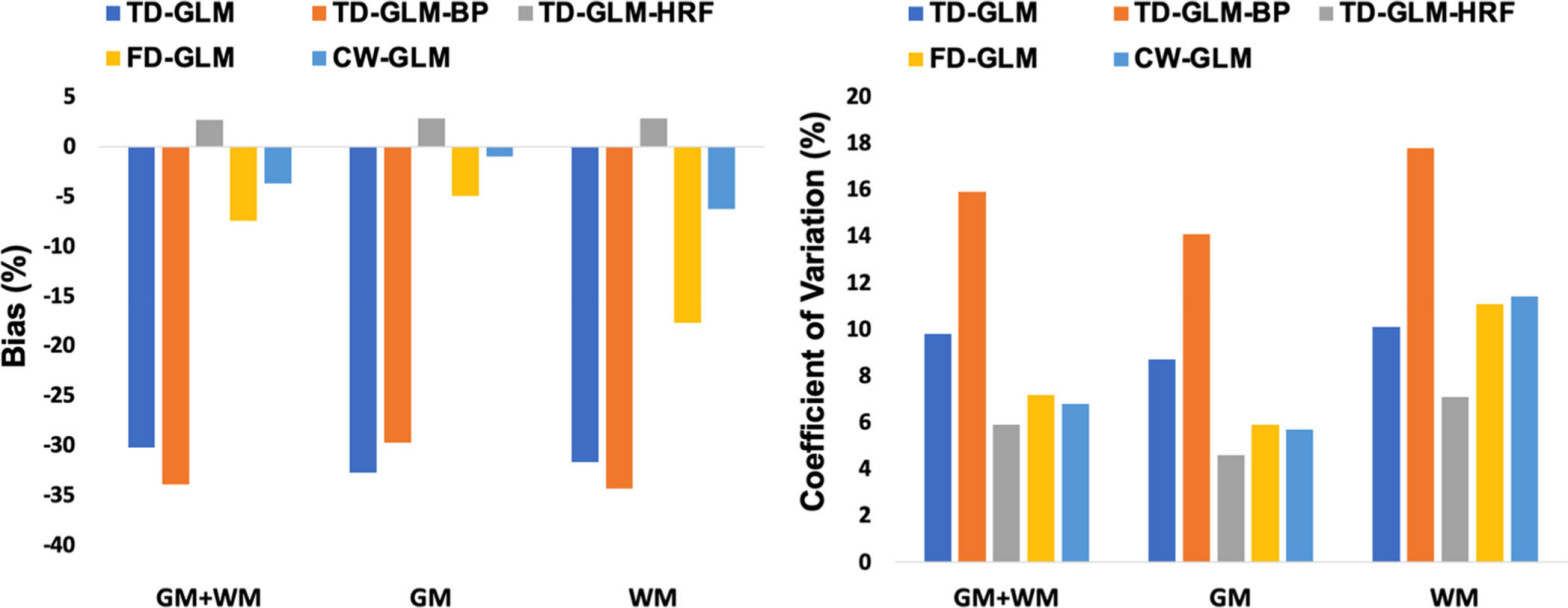
Bland Altman comparison of bias (left panels) and coefficient of variation (right panels) among all techniques for resting CVR and the sinusoidal CVR calculated from CW-GLM. Gray matter, white matter, and combined analyses are depicted.

In the Monte-Carlo simulation, the CW-GLM outperform all of the other methods for calculating the true CVR value. As a result, it makes sense to use CVR calculated from CW-GLM of the sine wave as the closest approximation to *in-vivo* ground truth. Fig. 11 represents the Bland-Altman comparison between sinusoidal CVR calculated from CW-GLM with resting CVR in other methods. The TD-GLM and TD-BP-GLM exhibit the same magnitude bias as observed in the Monte Carlo simulations; moderate bias is observed in FD-GLM, while TD-HRF-GLM and CW-GLM exhibit the least bias. Coefficient of variation between the methods are summarized for whole brain, gray matter and white matter in Fig. 11 (right). Complete Bland Altman analyses are also provided in Table 2(b). The coefficient of variation across techniques followed a pattern fairly similar to the in-silico validation, with TD-HRF-GLM exhibiting the best overall performance, closely followed by CW-GLM and FD-GLM.

### 3.6. Comparison between CVR and ALFF-based measurement

**Fig. 12.**
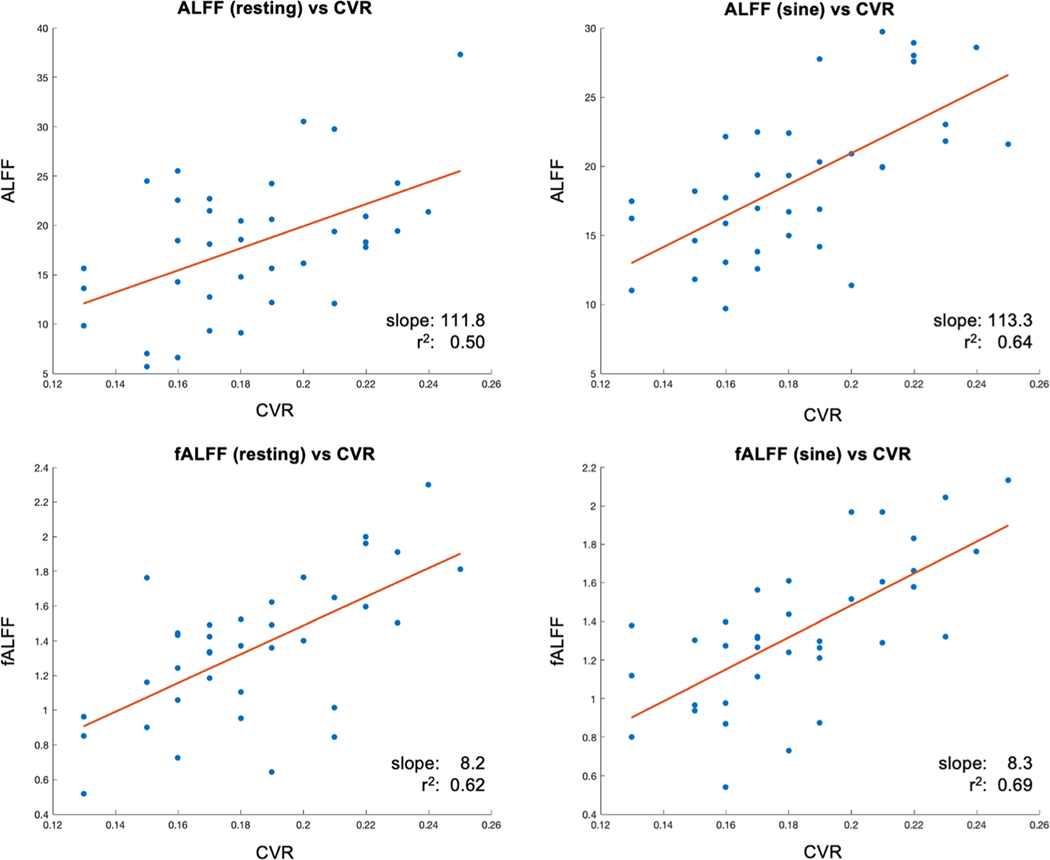
The correlation analysis among the global ALFF, fALFF, and CVR measurement.

Amplitude of low-frequency fluctuation (ALFF) or fractional amplitude of low-frequency fluctuation (fALFF) is a surrogate measurement of CVR from resting state fMRI. Since ALFF and fALFF are calculated from the power spectrum of fMRI signals and have no CO_2_ measurement to calibrate the signal power, they have different units from CVR and are not directly comparable. While the scale factor linking ALFF and fALFF to CVR is patient-specific, one still can compare the spatial similarity of ALFF and fALFF to CVR using correlation analysis using the sinusoidal CW-GLM as the benchmark. Fig. 12 shows the scattergram of the correlation analysis among the global ALFF, fALFF, and CVR measurement. The low frequency band chosen for ALFF and fALFF calculation is 0.01 – 0.08 Hz (Pinto et al., 2021). The result indicates that fALFF has a stronger correlation with CVR than ALFF. Additional voxel-wise r^2^ value is reported in Supplemental Table 1.

### 3.7. Coherence profile

**Fig. 13.**
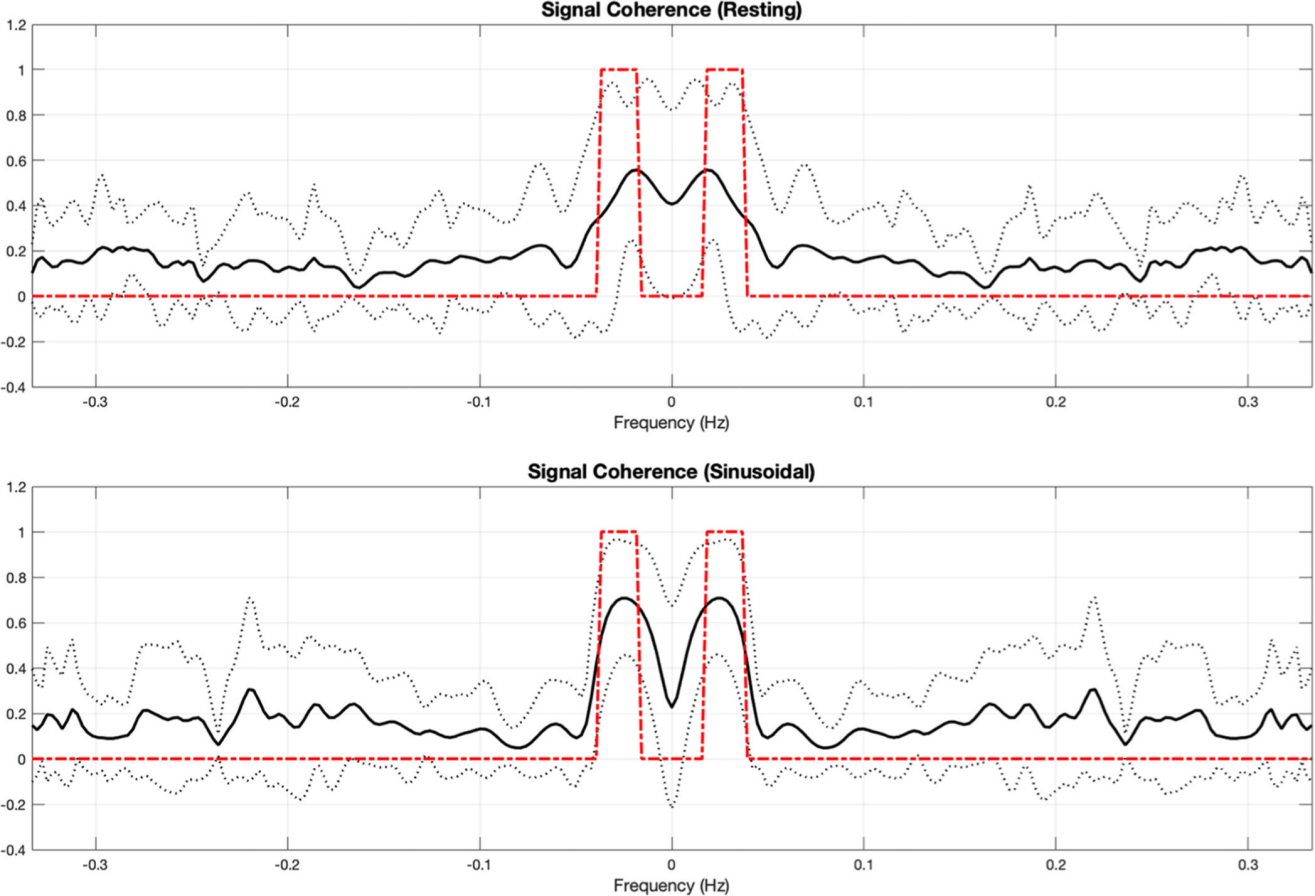
The average global signal coherence plot between end-tidal CO_2_ and BOLD signals from 36 subjects (black solid curve), the 95 % confidence interval (dotted curve), and the band-pass filter from 0.02 Hz to 0.04 Hz (dashed curve). The top plot is generated from signals recorded under resting state, and the bottom plot is generated from signals recorded under sinusoidal stimulus.

Insights into the contribution of bandpass filtering to TD-GLM and FD-GLM can be gained by examining the average coherence spectra between the CO_2_ and BOLD signals (Fig. 13), calculated separately for the resting (top panel) and sinusoidal (bottom panel). Despite the dramatic difference in the bandwidth of the CO_2_ stimuli, the coherence spectra for resting and sinusoidal signals are remarkably similar, with the power concentrated between 0.01 – 0.05 Hz. The 95 % confidence intervals in this region are broad, suggesting significant intersubject variability. Fig. 13 compares the overlap between coherence-weighting (solid line) and the band-pass filter recommended by Liu and colleagues (Liu et al., 2017). There is considerable overlap, but the “passband” is broader for the coherence spectrum, particularly with respect to the lower frequency cutoff.

**Fig. 14.**
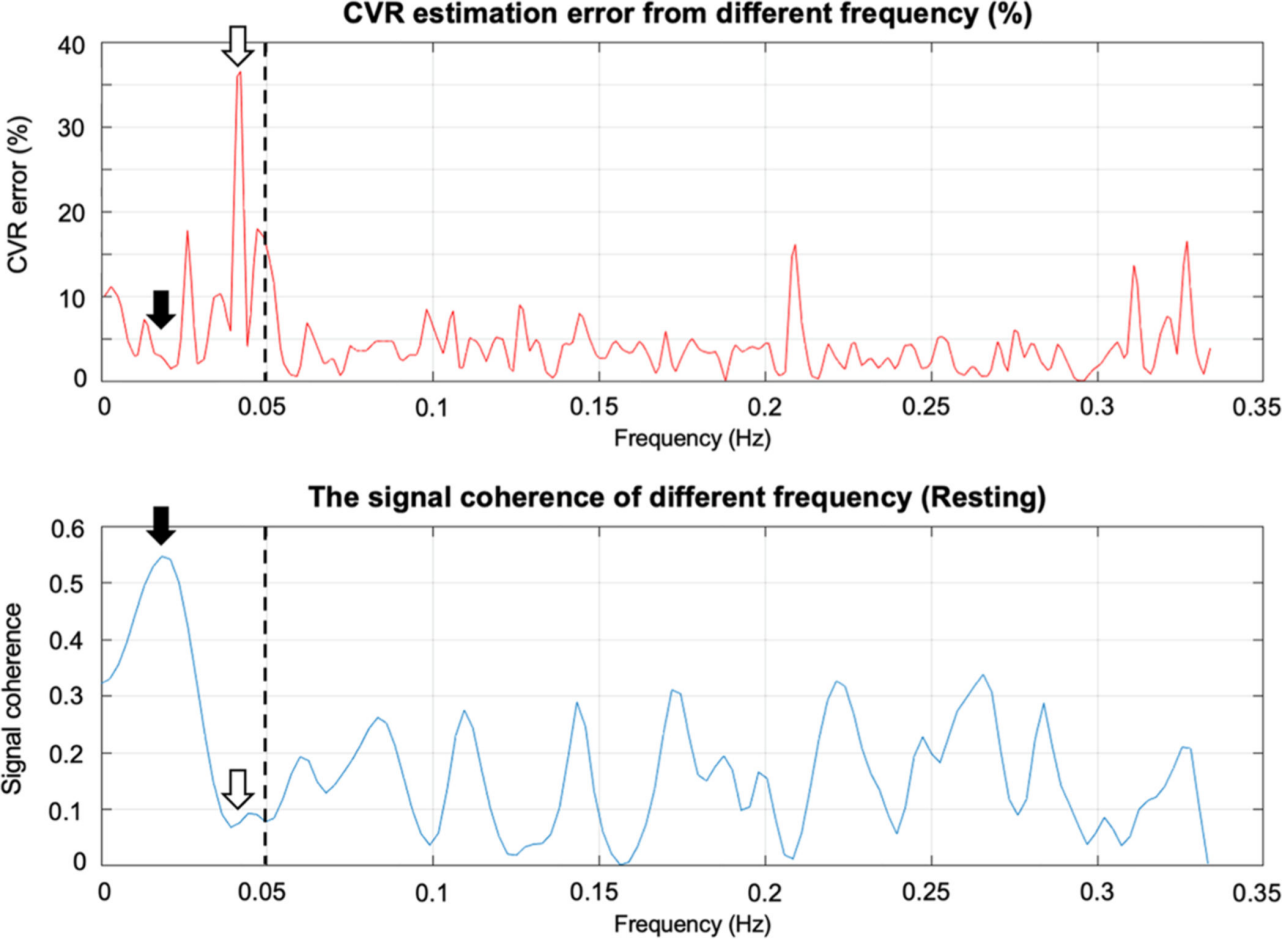
The CVR estimation error plot when using single frequency to calculate the CVR (top), and the corresponding signal coherence between end-tidal CO_2_ and BOLD signals (bottom). The error is calculated from synthetic data generated from Monte Carlo simulation with ground truth CVR. The vertical dash line indicates the frequency band of interest (0~0.05 Hz). The solid arrow points out the highest signal coherence and the lowest CVR estimation error, while the unfilled arrow points out the lowest signal coherence and the highest CVR estimation error.

It is possible to estimate CVR and delay from any single frequency, although this approach is only “optimal” for a pure sinusoidal CO2 stimulus. The CVR estimation errors using individual frequency components are shown in Fig. 14 (top) and compared to the corresponding signal coherence (bottom). The errors are calculated from synthetic data generated from the Monte Carlo simulation, and reported as percentages. Not surprisingly, estimation error varies inversely with coherence; errors above 15 % have coherence below 0.3, while coherence weights above 0.5 select frequencies with errors less than 5 %.

### 3.8. Alternative choices of the hemodynamic response function

**Fig. 15.**
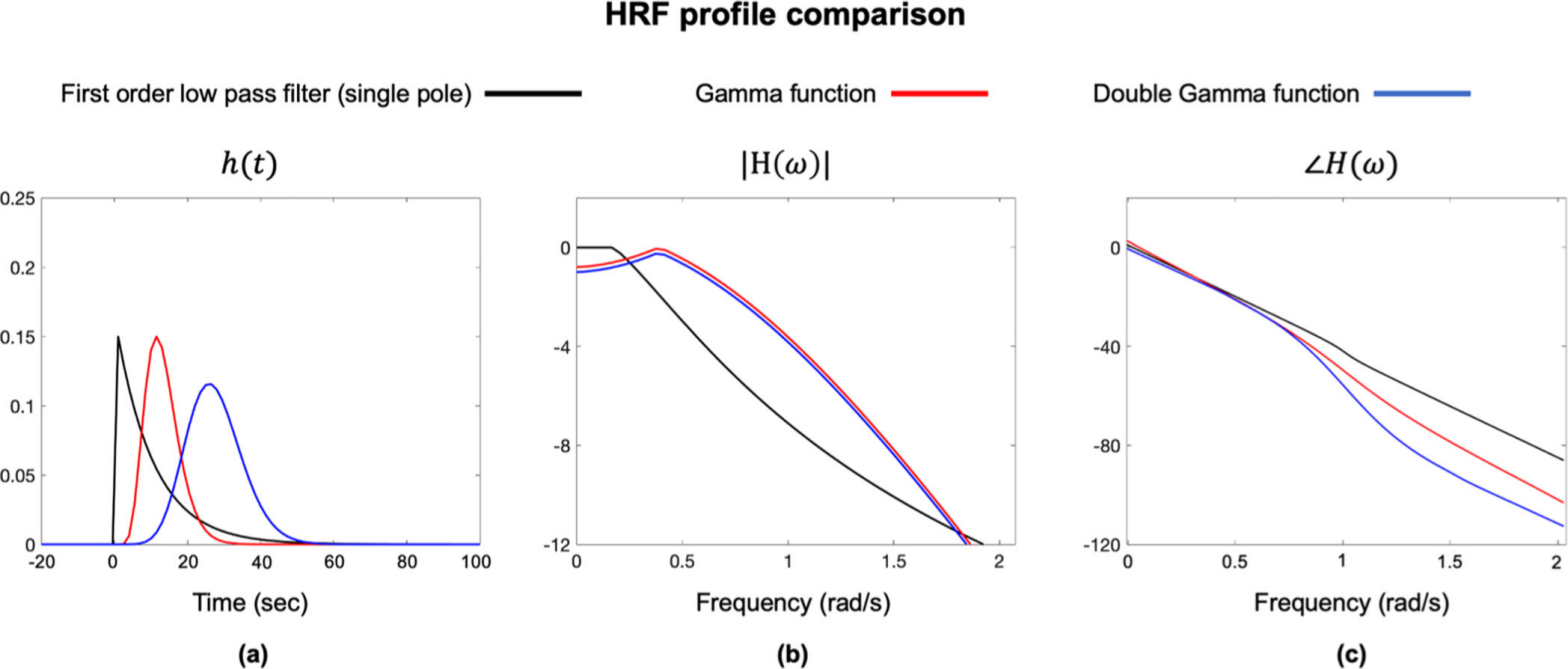
The profile of the different HRFs: (a) the time domain representation of h(t); (b) the magnitude of H(ω); (c) the phase of H(ω).

In our study, we model the h(t) as a first-order low-pass filter, while previous work has used a gamma function (Prokopiou et al., 2019) or a double-gamma function (Golestani et al., 2015) to model the brain’s HRF to CO_2_. The single pole low pass filter has one degree of freedom, the gamma function has two degrees of freedom, and the double gamma has six degrees of freedom, making the single pole model easier to estimate but less flexible. The profile of different HRFs are plotted in Fig. 15. The parameters for the gamma and double gamma function were estimated from representative examples (Golestani et al., 2015; Hossein-Zadeh et al., 2003; Prokopiou et al., 2019). According to the frequency representation, all three HRFs have similar pass bands except that the single pole passband extends to zero frequency, while the two gamma functions HRF’s attenuate frequencies below 0.06 Hz (time-constants longer than 16 s). Additionally, the gamma and double gamma HRFs have slightly higher cutoff frequencies, which is consistent with a faster response to CO_2_ changes. For the phase response, the gamma’s family have more nonlinearity than the single-pole low-pass filter, which refers to the more time shift. In contrast, the CW-filter attenuates frequencies below 0.01 (time constants longer than 100 s). Note that the BOLD signal power in this region can vary with the signal pre-processing. For example, Golestani uses high pass filtration with a cutoff of 0.01 Hz.

## 4. Discussion

### 4.1. General

CVR calculation is most commonly performed in the time-domain, where signal alignment is important for accurate performance (Liu et al., 2019). This is relatively straightforward for highly structured CO_2_ signals, but more challenging for resting state data. Many strategies have been proposed to optimize alignment (Donahue et al., 2016; Liu et al., 2017, 2020; 2021; Tong et al., 2011), with each method having pros and cons. Performing CVR calculation in frequency space obviates the need for signal alignment, with CVR maps coming directly from magnitude spectra and delay maps from phase shifts. In this manuscript, we demonstrate that frequency domain methods provide robust CVR estimates for both exogenous and endogenous CO_2_ stimuli. We also demonstrate that signal-coherence between CO_2_ and BOLD signals can be used as a matched filter to enrich contributions with higher contrast-to-noise for CVR measurement. Lastly, we demonstrate that adding a population-based model of the brain HRF could significantly improve TD-GLM.

We are not the first to consider CVR calculation in the Fourier domain (Blockley et al., 2011; 2017; Duffin et al., 2015), however, we are the first to evaluate this approach in resting state BOLD data and to exploit coherence-weighting to filter physiological noise. We used two criteria to compare performance among techniques. Firstly, we used data-driven Monte Carlo simulations to generate BOLD signals having ground truth values for both sinusoidal and resting stimuli. Secondly, we demonstrated that frequency domain methods yielded nearly identical performance from resting state BOLD and from a powerful CO_2_ challenge.

From the Monte-Carlo simulations, both frequency domain methods outperform time-domain methods for sine-wave CVR estimation. This intuitive result reflects the SNR enhancement from Fourier transformation of the narrow band EtCO_2_ signal. However, the EtCO_2_ spectrum is more broadband, yielding less SNR enhancement in the frequency domain. Coherence-weighting improved frequency domain performance (CW-GLM was better than FD-GLM), but did not outperform TD-GLM-HRF in the simulated data.

No ground truth exists for the real-world data, but the CVR estimates from the sinusoidal data have very high contrast to noise compared with resting state data allowing them to serve as reference data. Based on the Monte-Carlo simulation results, one would logically believe that the sine CVR/delay calculated by CW-GLM is the closest to “ground truth” for the *in-vivo* measurements. These data mirror the Monte-Carlo simulations very closely, with the relative 95 % confidence intervals TD-GLM-HRF <CW-GLM < FD-GLM < TD-GLM < TD-GLM-BP.

The astute reader might balk at the poor quality of the unfiltered TD-GLM results for the resting state data. Resting state data can be markedly improved by including coached-breathing maneuvers that introduce more natural end tidal CO_2_ fluctuations and improve signal to noise (Kastrup et al., 2001); these were not performed in this study. In fact, for approximately 50 % of the resting state acquisition, the RespirAct was actively targeting the end tidal CO_2_ concentration to the resting state determined over the first few minutes of the BOLD acquisition. Since the RespirAct achieves a targeting accuracy of +/− 1 torr (RespirAct, 2023), the resting state data in this study represent nearly worst-case SNR (the only thing worse would be end-tidal targeting for the entire acquisition). While our resting state data exaggerate the improvements one would observe in the absence of end-tidal CO_2_ targeting or coached breathing, it demonstrates the impressive ability of both TD-GLM-HRF and CW-GLM methods for EtCO_2_ signals with very poor CNR.

**Fig. 16.**
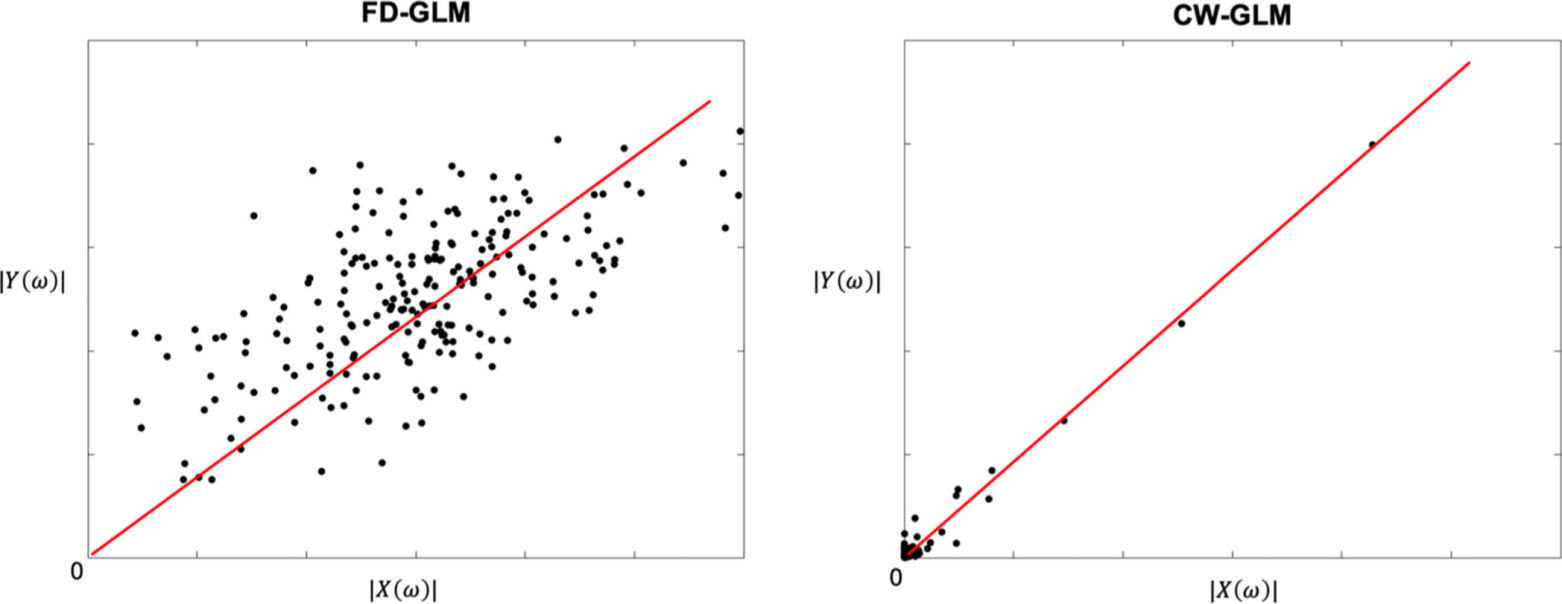
The scattergram for FD-GLM and CW-GLM graphical interpretations.

The concept of modeling the brain as a linear system is not novel (Bright and Murphy, 2013; Liu et al., 2017), nor even is the use of coherence to select a good frequency to calculate CVR (Duffin et al., 2015). But prior analyses stopped with the usage of sinusoidal CO_2_ challenges (Blockley et al., 2011, 2013) and generalization to arbitrary stimuli was not appreciated. FD-GLM and CW-GLM have very intuitive graphical interpretations. If one considers a scattergram of |Y(ω)| versus |X(ω)|, FD-GLM and CW-GLM represent the best fit line through the data (shown in Fig. 16). When the line is constrained to pass through the origin, the slope is CVR. For FD-GLM, all frequencies contribute equally to the slope calculation (uniform weighting function), even though some of those frequencies are heavily contaminated by physiological noise from spontaneous brain activity and cerebral autoregulation. The CW-GLM uses signal coherence to weight the linear fitting. A signal with coherence of one lies on the CVR line with zero residual and is weighted heavily in the fitting. In the case of an infinite sine-wave, all other frequencies would be completely rejected and CVR would simple represent the ratio of |Y(ω)|/|X(ω)| at a single point. However, in resting state, no single dominant frequency exists. Coherence-weighting acts like a matched filter to emphasize those frequency components in the BOLD signal that can be explained by fluctuations in CO_2_.

Simple TD-GLM and TD-GLM-BP both model the brain’s HRF as an all-pass filter with linear phase response. These methods failure to account for the frequency response of the brain to CO_2_ fluctuations causes resting CVR measurements to severely underestimate true CVR. A fairly well-cited review (Liu et al., 2019), makes no mention of HRF correction. There are also other CVR measurements without mentioning the HRF correction (Dlamini et al., 2018; Nishida et al., 2019; Taneja et al., 2019; Vestergaard and Larsson, 2019). However, prefiltering the EtCO_2_ signal using a population-based estimate of the HRF, prior to TD-GLM calculation, removed the bias and provided equal or better performance to the frequency domain methods. In fact, most of the improvement performance of the frequency domain methods in resting state, relative to TD-GLM and TD-BP-GLM, resulted from incorporation of a realistic HRF rather than inherent advantages of performing the calculations in the frequency domain.

### 4.2. Association between coherence-weighting and band-pass filtering

Bandpass filtering between 0.02 Hz and 0.04 Hz has been used to improve the appearance of CVR maps. Choice of this passband is empiric (Liu et al., 2017). Bandpass filtering improves the appearance of CVR maps as well as time-delay quantification implying that the passband frequencies have high spatial correlation. Despite the improved appearance, CVR estimation is significantly degraded with respect to ground truth (Table 1, 2a, 2b).

The profile of coherence-weighting (shown in Fig. 14) also has a band-pass structure, which the larger weights roughly fall into the frequency band from 0.01 Hz to 0.05 Hz. The broader passband observed in the average coherence filter likely reflects intersubject variability in the “optimal” bandpass filter cutoffs. This hypothesis is supported by the broad 95 % confidence intervals in the ensemble average coherence spectrum. That is, no single fixed passband is perfect for all subjects and CW-GLM excels by “matching” the filter coefficients individually. We postulate that the intersubject variability in coherence is why fixed bandpass filtering increases CVR measurement error. Patient-specific matching may also be important because some sources of physiological noise, such as cerebral autoregulation, map squarely into the passband (Pinto et al., 2021). Since this spectral component is not tightly correlated to end-tidal CO_2_, coherence weighting will de-emphasize this physiological noise contribution. Both the bandpass and coherence-weighting are linear filtering operations, which reduce signal complexity, lowering the degrees of freedom (or entropy) of the signal, thereby improves statistical robustness.

### 4.3. Limitations

By applying GLM (in either time or frequency domain) to estimate CVR, we assume that the brain’s hemodynamic response function is linear and time-invariant. While this represents current-state-of-the-art in CVR measurement, and is true for the magnitude of CO_2_ changes used in most studies, any GLM neglects any non-linearities in cerebrovascular system.

For simplicity, we used a fixed, population-based, α estimate of 0.3 s^−1^ for gray matter and 0.12 s^−1^ for white matter to calculate CVR and delay maps for the frequency techniques. This parameter may vary among subjects as well as regionally, however our simplified approach still yielded highly accurate results. Nonetheless, intersubject variation in temporal response (e.g. the α term in Eq. (9)) could potentially be an important independent physiological prognosticator (Christen et al., 2015; Donahue et al., 2016; Thomas et al., 2014; Yezhuvath et al., 2009). In highly structure signals, it is possible to estimate the HRF from the data. For example, with a sinusoidal signal, it is straightforward to estimate α from the phase and use it to properly scale the magnitude spectral coefficients.

In our evaluation of the performance, the resting TD-GLM-BP and FD-GLM-BP relative to their sinusoidal equivalents will be slightly exaggerated because the sine wave is outside the passband. The effect is relatively modest. Lowering the bandpass cutoff from 0.2 to 0.16 would lead to a signal increases of 28 %, 48 % and 72 % for filter orders of 4, 6, and 8, respectively. The purpose of not modifying the filter frequencies to include the sinusoid frequency was to make a fair, systematic comparison with existing methods. In general, we do not know the optimal passband. Hence, using signal coherence as subject-specific bandpass filters is intuitively satisfying. Note that the agreement between resting and sinusoidal CVR measurements was higher for CW-GLM than either TD-GLM-BP and FD-GLM-BP, despite the artificial boost from the sinusoid lying outside the passband.

The participants in this study were all young, while the temporal SNR (tSNR) and signal change will be lower in old population. Inclusion of elderly subjects would simply lower the tSNR and degrade the performance of all methods. However, the relative performance of the methods should not change. If anything, the justification for the FD-GLM and CW-GLM would be increased because temporal alignment eventually becomes impossible when tSNR is sufficiently low.

For strong stimuli such as the sine wave, coherence weighting of the phase response can be used to construct accurate delay maps. However, for the resting state data, the phase SNR was too low to construct robust delay maps, even though CW-GLM outperformed the other methods. Coached-breathing would improve the phase SNR by increasing end-tidal CO_2_ contrast (Bright and Murphy, 2013; Pinto et al., 2016; 2021) and would likely be needed to obtain good delay maps from resting data.

To reconstruct delay maps accurately from signal phase, cerebral hemodynamics need to be the dominant source of signal delay. Additional signal delays include arterial transit time and delays from the end-tidal CO_2_ recording devices. Arterial transit times of 1–2 s are too short to significantly confound delay estimates. Gas sampling delays were also short in the present study but can be much longer if the CO_2_ sampling motor is weak or sampling lines are long. In this scenario, gas sampling delays must be characterized by calibration studies and the subsequent linear phase shift removed before calculating frequency domain delay estimates.

TD-GLM can produce voxels with negative CVR value, particularly in deep watershed areas. In frequency-based methods, these will map to positive CVR values with a half-period phase shift. This is not artifact but simply the mathematical representation of an out-of-phase signal. The vascular bed is not a purely resistive system, leading to phase dispersion (that can be as high as 180^°^) between superficial and deep structures.

The proposed method is agnostic to the contrast used to detect vascular reactivity, which could include vascular space occupancy (VASO), or arterial spin labeling (ASL). Application of this method to these techniques is beyond the scope of this manuscript but should be feasible.

The proposed method is also agnostic to the form of the input signal. In fact, in this study, we have use two polar extremes in spectral content to interrogate the brain’s transfer function. The sinusoidal stimulus is broad band in the time domain and narrow band in the frequency domain. The resting state signal has the opposite characteristics. Despite this, we were able to generate highly concordant CVR maps.

Time-domain and frequency domain analyses may be complementary, and we did not examine every combination. For example, one could consider using coherence weighting in lieu of a fixed bandpass filter before performing TD-GLM. Inherently, the time-domain and frequency domain contain the same information and the relative advantages/disadvantages of processing in either may depend on the application.

Given the similarity among the different HRF’s (Fig. 16), we cannot claim superiority of one over another. Our goal was to keep the modeling as simple as possible to still yield reasonable CVR and delay estimates. That is why we chose a single, population-based, tissue-invariant alpha rather than trying to estimate it from the data. The point of this paper is that even this simplistic approach can achieve accurate and robust CVR measurements. However, future work exploring alternative and voxel-specific HRF parameters is warranted.

## 5. Conclusion

CW-GLM complements current GLM based CVR estimation approaches. Fourier transformation separates the CVR and delay estimation into the spectral magnitude and phase, respectively, obviating the need for temporal alignment. Coherence weighting suppresses unimportant signals, which improves the frequency-domain CVR estimates by limiting contributions from noncontributory frequencies. We demonstrated that CW-GLM has superior performance to time-domain methods from sinusoidal acquisitions while it yields comparable performance from resting state signals. Although sinusoidal stimuli are not often used, periodic stimuli such as trained breath-holding or coached deep-breathing are common; this will concentrate power in the Fourier domain and should improve the relative performance of CW-GLM. In this manuscript, we also demonstrate that incorporating a fixed, population-based HRF model in time-domain CVR assessments yielded slightly more robust values than the CW-GLM for our ETCO_2_-clamped resting BOLD signals. Thus, the “optimum” CVR method depends on CO_2_ stimulus/maneuvers, but can be easily assessed by Monte-Carlo simulation. Delay estimation requires greater CO_2_ fluctuation for accurate measurements than CVR estimation and was challenging for both time and frequency domain techniques in the present resting-state paradigm.

So, what this means is that one can substitute resting state for structured stimuli if CVR is the primary parameter of interest. However, one cannot ignore that fact that delay maps contain important physiological information about vascular response dynamics. Estimating delay is therefore not a “methods” problem but a SNR problem, so it is critical to put structure into the end-tidal CO2 signal through some means if delay estimation is desired. However, high quality, accurate CVR maps were obtainable from resting state data in all subjects using TD-GLM-HRF and CW-GLM methods. These observations are important for clinical, pediatric, and dementia-related applications, where compliance with even simple respiratory tasks is often poor, and stressful and expensive apparatus are infeasible.

## Supplementary Material

1

## Figures and Tables

**Table 1:** The quantitative evaluation for different CVR estimation methods in 10,000 synthetic data. The results of Monte Carlo simulation are reported in Bland-Altman bias and variance (%), *t*-test, and mean absolute error (%). (BP: with band-pass filter; HRF: with hemodynamic response function; T/F: the ratio of the T and F statistics.).

Monte Carlo Simulation						
Method	Bias and Variance (%)	T/F	Mean absolute error (%)	Bias and Variance (%)	T/F	Mean absolute error (%)

	**Resting CVR (gray Matter)**			**Sinusoidal CVR (gray Matter)**		
**TD-GLM**	−28.3 ± 6.5	−4.4/0.6	28.3 ± 6.5	−31.7 ± 2.9	−10.9/0.6	31.7 ± 2.9
**TD-GLM-BP**	−29.2 ± 8.7	−3.4/0.9	29.3 ± 8.6	−33.9 ± 3.3	−10.3/0.5	33.9 ± 3.3
**TD-GLM-HRF**	2.2 ± 4.6	0.5/1.2	3.9 ± 3.2	2.4 ± 4.3	0.6/1.3	3.9 ± 2.9
**TD-GLM-BP-HRF**	5.0 ± 12.8	0.4/1.9	10.3 ± 9.0	−2.4 ± 5.0	−0.5/1.1	4.5 ± 3.3
**FD-GLM**	−5.1 ± 5.0	−1.0/1.1	5.7 ± 4.3	−1.1 ± 0.9	−1.2/1.0	1.2 ± 0.8
**FD-GLM-BP**	8.0 ± 12.6	0.6/1.9	11.2 ± 9.8	−0.6 ± 5.1	−0.1/1.2	4.1 ± 3.0
**CW-GLM**	−1.7 ± 5.3	−0.3/1.2	4.1 ± 3.8	−0.4 ± 0.7	−0.6/1.1	0.7 ± 0.5
	**Resting CVR (White Matter)**			**Sinusoidal CVR (White Matter)**		
**TD-GLM**	−27.9 ± 7.4	−3.8/0.5	27.9 ± 7.4	−31.7 ± 4.4	−7.2/0.5	31.7 ± 4.4
**TD-GLM-BP**	−28.5 ± 12.1	−2.4/0.9	28.8 ± 11.4	−33.7 ± 5.6	−6.0/0.5	33.7 ± 5.6
**TD-GLM-HRF**	2.3 ± 6.9	0.3/1.3	5.6 ± 4.7	2.4 ± 6.7	0.4/1.2	5.6 ± 4.3
**TD-GLM-BP-HRF**	6.0 ± 18.0	0.3/2.0	14.0 ± 12.7	−2.1 ± 8.5	−0.2/1.2	7.0 ± 5.3
**FD-GLM**	−17.4 ± 9.0	−1.9/1.1	17.4 ± 8.9	−3.0 ± 1.4	−2.1/1.0	3.1 ± 1.4
**FD-GLM-BP**	12.4 ± 18.7	0.7/1.8	16.3 ± 15.4	− 0.3 ± 8.7	−0.1/1.2	6.9 ± 5.2
**CW-GLM**	−6.4 ± 8.7	−0.7/1.2	8.0 ± 7.2	−0.9 ± 1.1	−0.8/1.1	1.2 ± 0.9
	**Resting CVR delay (gray Matter)**			**Sinusoidal CVR delay (gray Matter)**		
**TD-GLM**	−0.3 ± 29.7	0.0/15.1	21.2 ± 9.5	0.1 ± 8.3	0.0/3.3	6.7 ± 2.2
**TD-GLM-BP**	0.1 ± 9.7	0.0/3.9	7.3 ± 2.7	−0.1 ± 5.0	0.0/2.3	4.2 ± 1.3
**TD-GLM-HRF**	−0.3 ± 24.3	0.0/11.6	16.7 ± 8.6	0.1 ± 8.2	0.0/3.3	6.2 ± 2.3
**TD-GLM-BP-HRF**	0.1 ± 10.1	0.0/4.0	7.9 ± 2.6	− 0.1 ± 5.5	0.0/2.4	4.3 ± 1.4
**FD-GLM**	−0.1 ± 9.7	0.0/3.9	7.1 ± 2.9	0.0 ± 4.8	0.0/2.2	3.7 ± 1.2
**FD-GLM-BP**	−0.1 ± 9.8	0.0/3.9	7.3 ± 2.8	0.0 ± 4.8	0.0/2.2	3.8 ± 1.3
**CW-GLM**	−0.1 ± 9.8	0.0/3.9	6.7 ± 2.2	0.0 ± 4.6	0.0/2.1	3.6 ± 1.1
	**Resting CVR delay (White Matter)**			**Sinusoidal CVR delay (White Matter)**	
**TD-GLM**	−0.3 ± 34.6	0.0/19.9	24.7 ± 10.3	0.1 ± 11.0	0.0/4.4	8.1 ± 3.1
**TD-GLM-BP**	0.2 ± 12.9	0.0/5.2	9.1 ± 4.1	− 0.1 ± 7.4	0.0/3.0	4.9 ± 2.7
**TD-GLM-HRF**	−0.3 ± 28.3	0.0/14.7	19.5 ± 9.1	0.1 ± 10.5	0.0/4.2	7.9 ± 2.8
**TD-GLM-BP-HRF**	0.2 ± 13.2	0.0/5.4	9.2 ± 4.3	− 0.1 ± 7.6	0.0/3.1	5.1 ± 2.7
**FD-GLM**	−0.2 ± 12.2	0.0/4.9	8.7 ± 3.7	0.1 ± 6.9	0.0/2.9	4.5 ± 2.6
**FD-GLM-BP**	−0.2 ± 12.5	0.0/5.1	8.7 ± 3.9	0.1 ± 7.1	0.0/2.9	4.6 ± 2.7
**CW-GLM**	−0.2 ± 11.4	0.0/4.6	8.2 ± 3.5	0.1 ± 6.8	0.0/2.8	4.5 ± 2.4

**Table 2a:** The Bland Altman analysis results of intra-method comparison between resting state and sinusoidal stimulus. The results are calculated from 36 subjects. (BP: with band-pass filter; HRF: with hemodynamic response function.).

Bland Altman analysis between the resting and sinusoidal CVR from each method									
Method	whole brain			gray matter			White Matter		
	Mean ± Std (%)	95 % Confidence Interval	p-value	Mean ± Std (%)	95 % Confidence Interval	p-value	Mean ± Std (%)	95 % Confidence Interval	p-value

TD-GLM	5.37 ± 12.29	[−18.72, 29.46]	0.013	5.20 ± 10.74	[−15.85, 26.25]	< 0.01	5.81 ± 10.43	[−14.63, 26.25]	< 0.01
TD-GLM-BP	5.17 ± 18.94	[−31.95, 42.29]	0.110	11.35 ± 15.56	[−19.15, 41.85]	< 0.01	5.83 ± 19.68	[−32.74, 44.40]	0.084
TD-GLM-HRF	−1.95 ± 8.71	[−19.02, 15.12]	0.188	0.55 ± 7.49	[−14.13, 15.23]	0.662	0.23 ± 9.72	[−18.82, 19.28]	0.888
FD-GLM	−6.76 ± 7.28	[−21.03, 7.51]	< 0.01	−4.28 ± 6.07	[−16.18, 7.62]	< 0.01	−15.07 ± 11.30	[−37.22, 7.08]	< 0.01
CW-GLM	−3.72 ± 6.76	[−16.70, 9.53]	< 0.01	−1.03 ± 5.67	[−12.14, 10.08]	0.283	−6.27 ± 11.36	[−28.54, 16.00]	< 0.01

**Table 2b:** The Bland Altman analysis results of inter-method comparison between resting CVR from each method and sinusoidal CVR from CW-GLM. The results are calculated from 36 subjects. (BP: with band-pass filter; HRF: with hemodynamic response function.).

Bland Altman analysis between the resting CVR from each method and sinusoidal CVR from CW-GLM									
Method	whole brain			gray matter			White Matter		
	Mean ± Std (%)	95 % Confidence Interval	p-value	Mean ± Std (%)	95 % Confidence Interval	p-value	Mean ± Std (%)	95 % Confidence Interval	p-value

TD-GLM	−30.24 ± 9.75	[−49.35, −11.13]	< 0.01	−32.68 ± 8.65	[−49.63, −15.73]	< 0.01	−31.71 ± 10.06	[−51.43, −11.99]	< 0.01
TD-GLM-BP	−33.90 ± 15.85	[−64.97, −2.83]	< 0.01	−29.72 ± 14.11	[−57.38, −2.06]	< 0.01	−34.35 ± 17.77	[−69.18, 0.48]	< 0.01
TD-GLM-HRF	2.65 ± 5.85	[−8.82, 14.12]	0.010	2.83 ± 4.63	[−6.24, 11.90]	< 0.01	2.81 ± 7.08	[−11.07, 16.69]	0.023
FD-GLM	−7.51 ± 7.15	[−21.52, 6.50]	< 0.01	−4.95 ± 5.94	[−16.59, 6.69]	< 0.01	−17.68 ± 11.08	[−39.40, 4.04]	< 0.01
CW-GLM	−3.72 ± 6.76	[−16.70, 9.53]	< 0.01	−1.03 ± 5.67	[−12.14, 10.08]	0.283	−6.27 ± 11.36	[−28.54, 16.00]	< 0.01

**Table 3:** The regional voxel-wise correlation analysis between resting and sinusoidal CVR from 36 subjects. p-value indicates the significant difference of the variance between GM and WM for different methods. (GM: gray matter; WM: white matter; BP: with band-pass filter; HRF: with hemodynamic response function.).

Method	Slope (GM)	Slope (WM)	p-value	R square (GM)	R square (WM)	p-value

**TD-GLM**	0.94 ± 0.19	0.83 ± 0.15	<0.01	0.69 ± 0.16	0.43 ± 0.12	<0.01
**TD-GLM-BP**	0.96 ± 0.12	0.92 ± 0.12	0.119	0.74 ± 0.13	0.46 ± 0.14	<0.01
**TD-GLM-HRF**	1.00 ± 0.10	0.98 ± 0.11	0.283	0.76 ± 0.14	0.44 ± 0.11	<0.01
**FD-GLM**	0.96 ± 0.13	0.89 ± 0.16	0.013	0.74 ± 0.12	0.44 ± 0.12	<0.01
**CW-GLM**	1.01 ± 0.07	0.99 ± 0.12	0.258	0.75 ± 0.11	0.45 ± 0.11	<0.01

**Table 4:** The regional voxel-wise correlation analysis between resting and sinusoidal delay from 36 subjects. p-value indicates the significant difference of the variance between GM and WM for different methods. (GM: gray matter; WM: white matter; BP: with band-pass filter; HRF: with hemodynamic response function.).

Method	Slope (GM)	Slope (WM)	p-value	R square (GM)	R square (WM)	p-value

**TD-GLM**	0.83 ± 0.17	0.79 ± 0.20	0.298	0.67 ± 0.16	0.52 ± 0.14	<0.01
**TD-GLM-BP**	0.82 ± 0.16	0.81 ± 0.17	0.626	0.71 ± 0.14	0.56 ± 0.13	<0.01
**TD-GLM-HRF**	0.84 ± 0.17	0.80 ± 0.19	0.267	0.69 ± 0.15	0.53 ± 0.14	<0.01
**FD-GLM**	0.87 ± 0.15	0.84 ± 0.18	0.423	0.75 ± 0.13	0.59 ± 0.12	<0.01
**CW-GLM**	0.88 ± 0.15	0.85 ± 0.17	0.496	0.76 ± 0.13	0.60 ± 0.12	<0.01

## Data Availability

Data will be made available on request.

## Data and code availability statement

Deidentified imaging data, simulations, and processing code used in this work will be made available to qualified researchers upon request on a case-by-case basis after obtaining approval from the CHLA regulatory authorities. The source code of the methods and demo are available on https://github.com/jwoodchla/Coherence-Weighted-GLM.
