# Supplementary material for: Improved cerebrovascular reactivity mapping using coherence weighted general linear model in the frequency domain
Source: Neuroimage. Author manuscript; Available in PMC 2024 Jan 28. (PMC10822713; doi:10.1016/j.neuroimage.2023.120448)
Supplement: 1 [file NIHMS1949325-supplement-1.docx]

**Supplementary Material**

**Additional CVR maps**


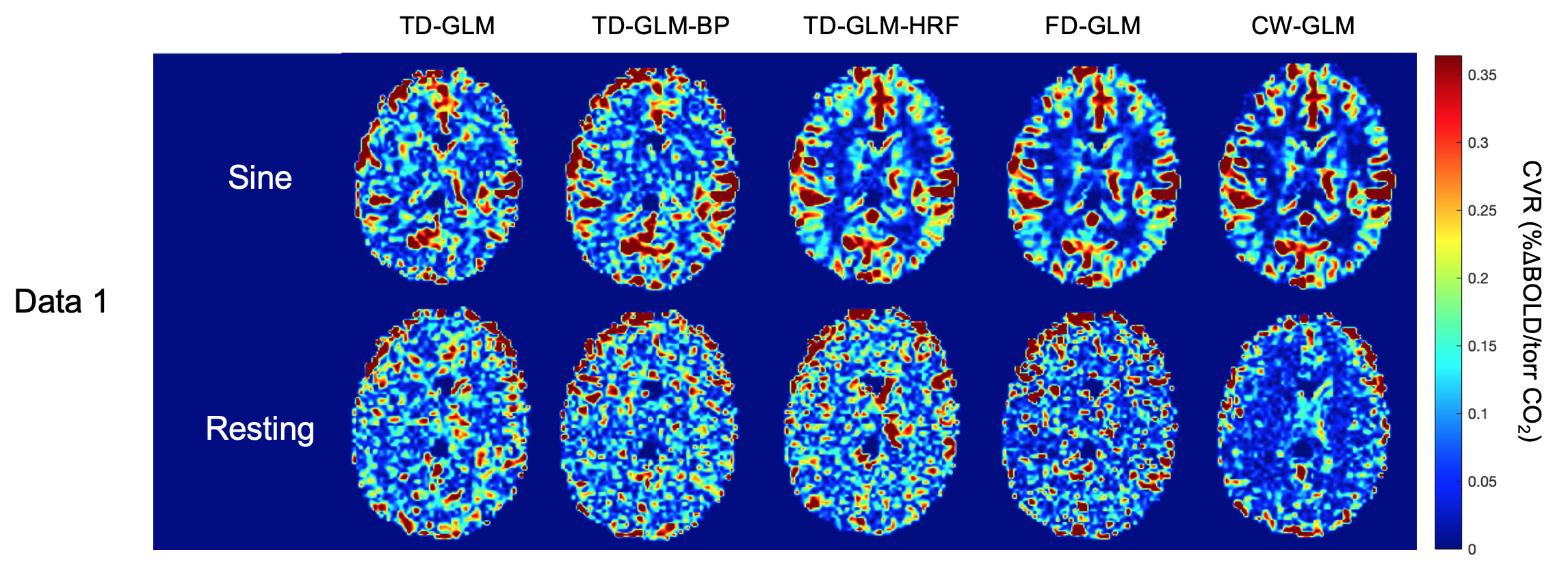

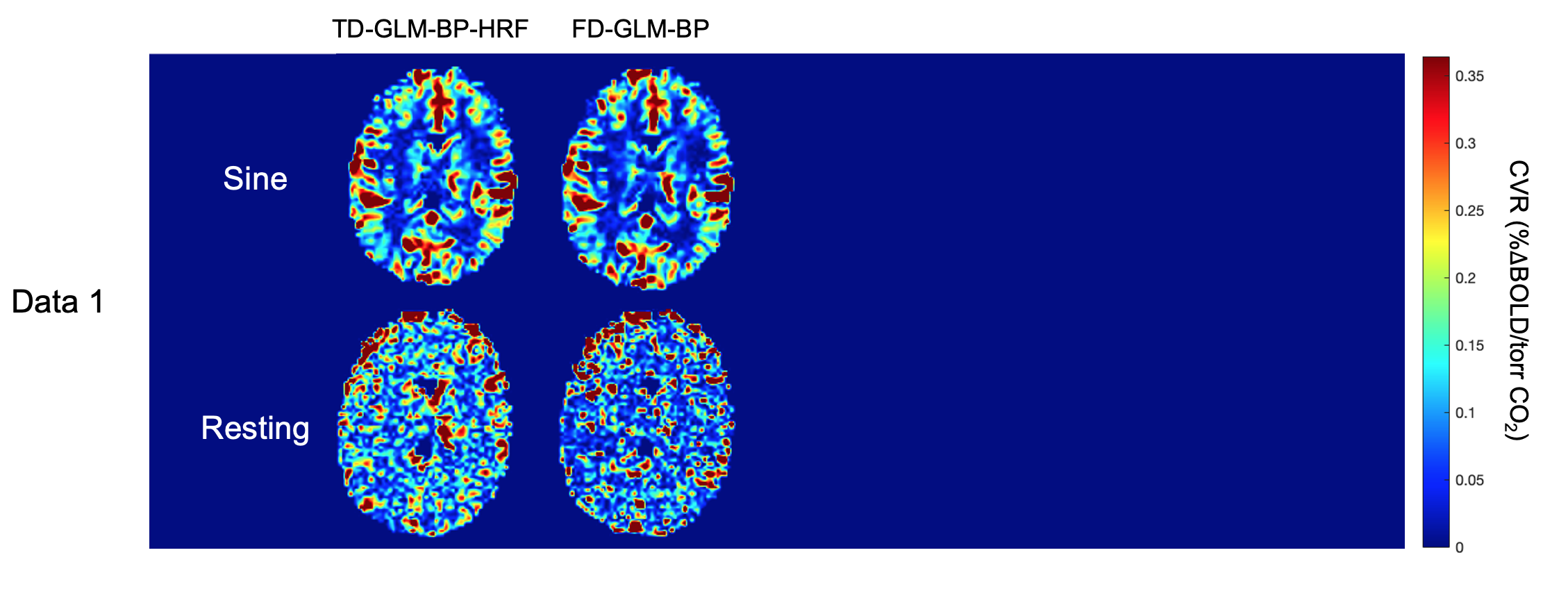

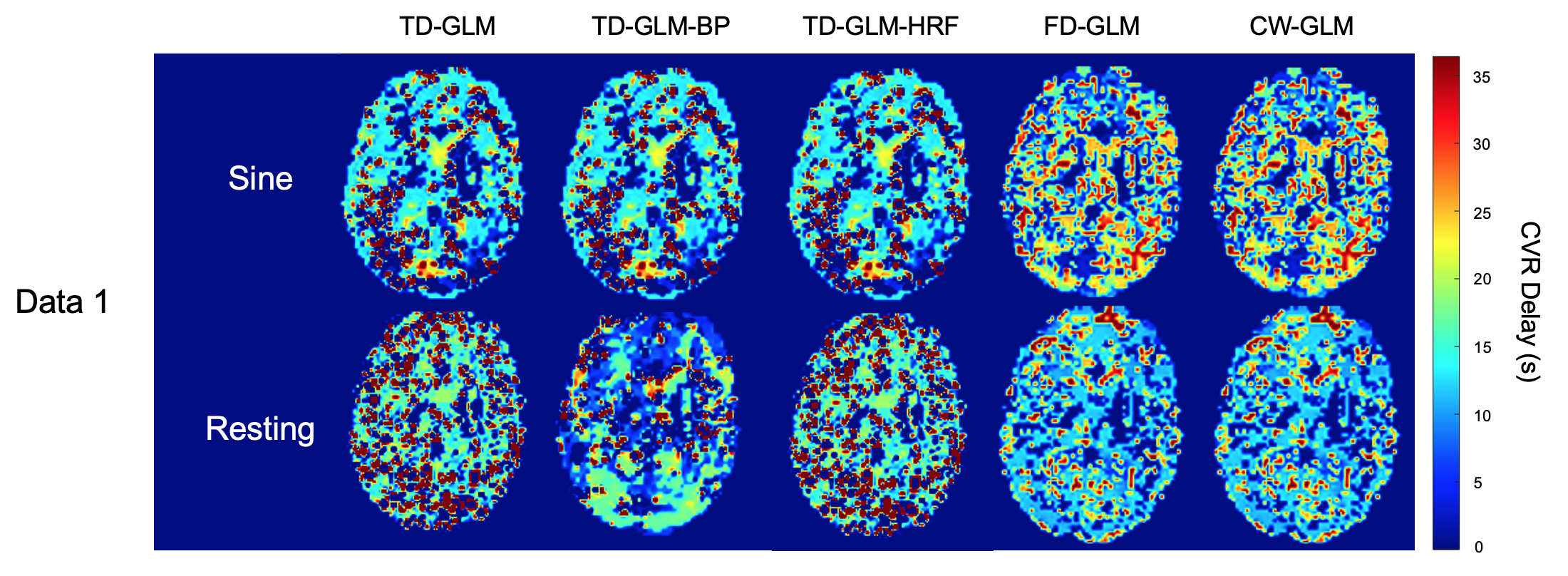

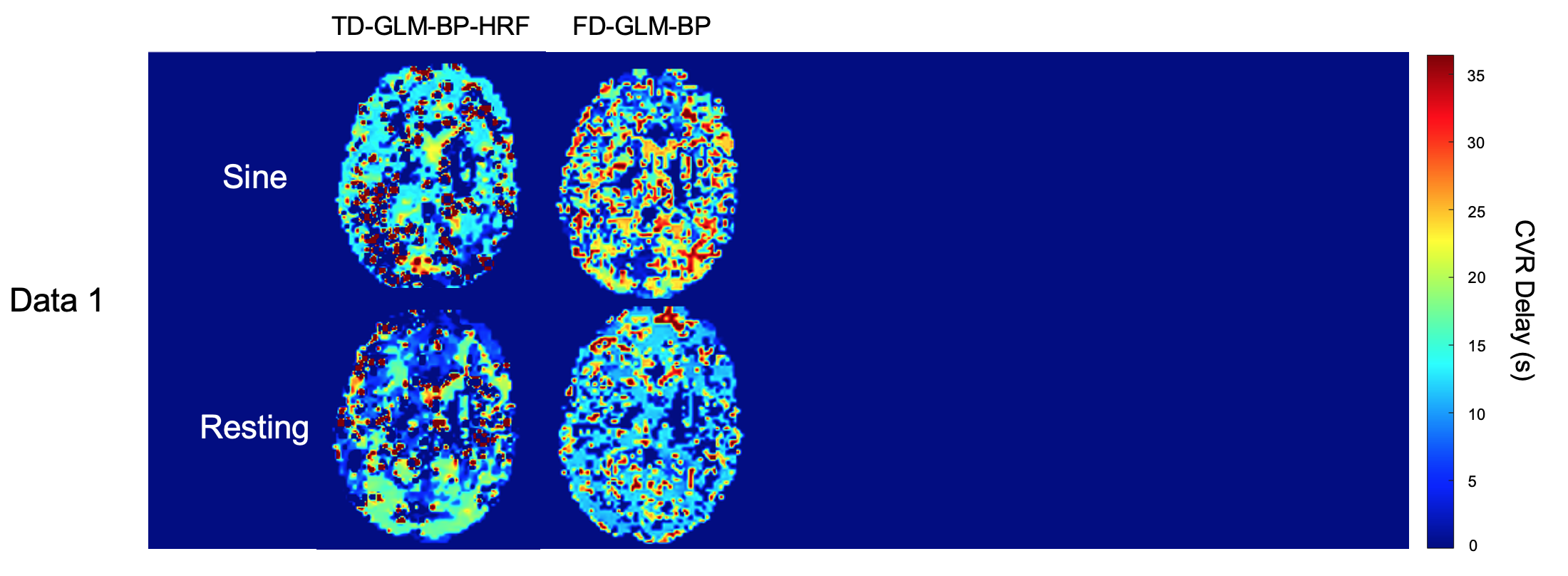


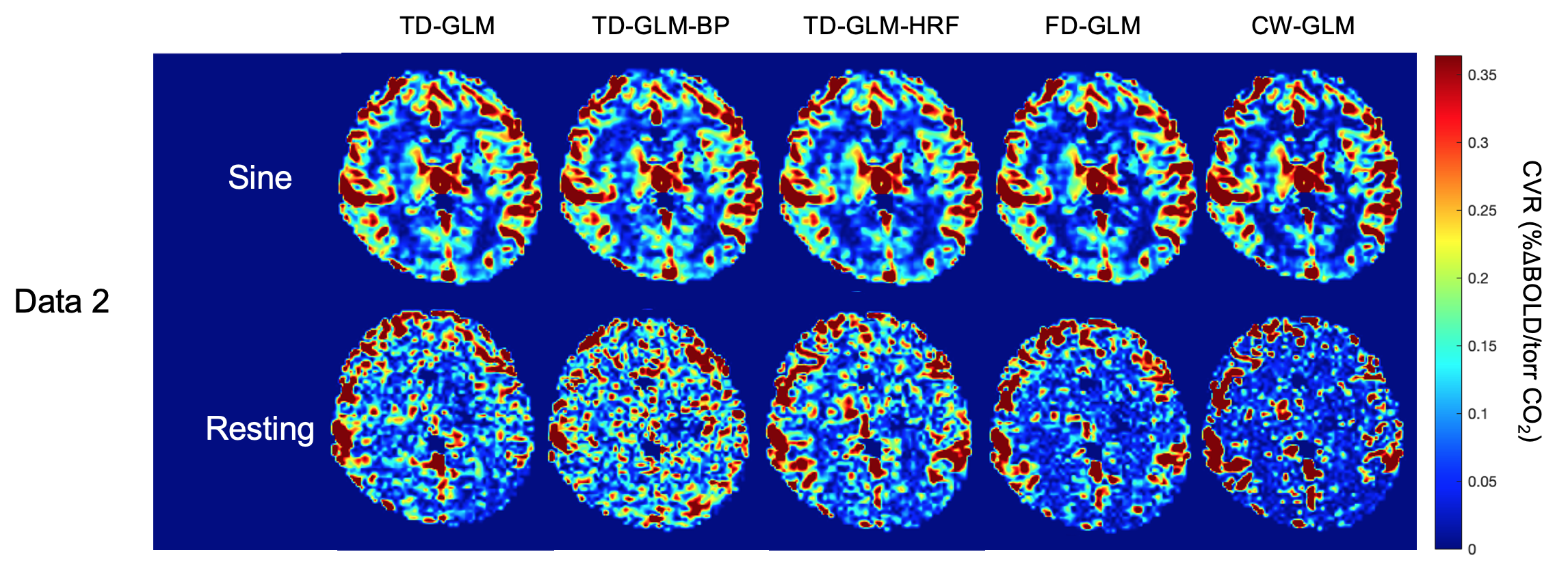

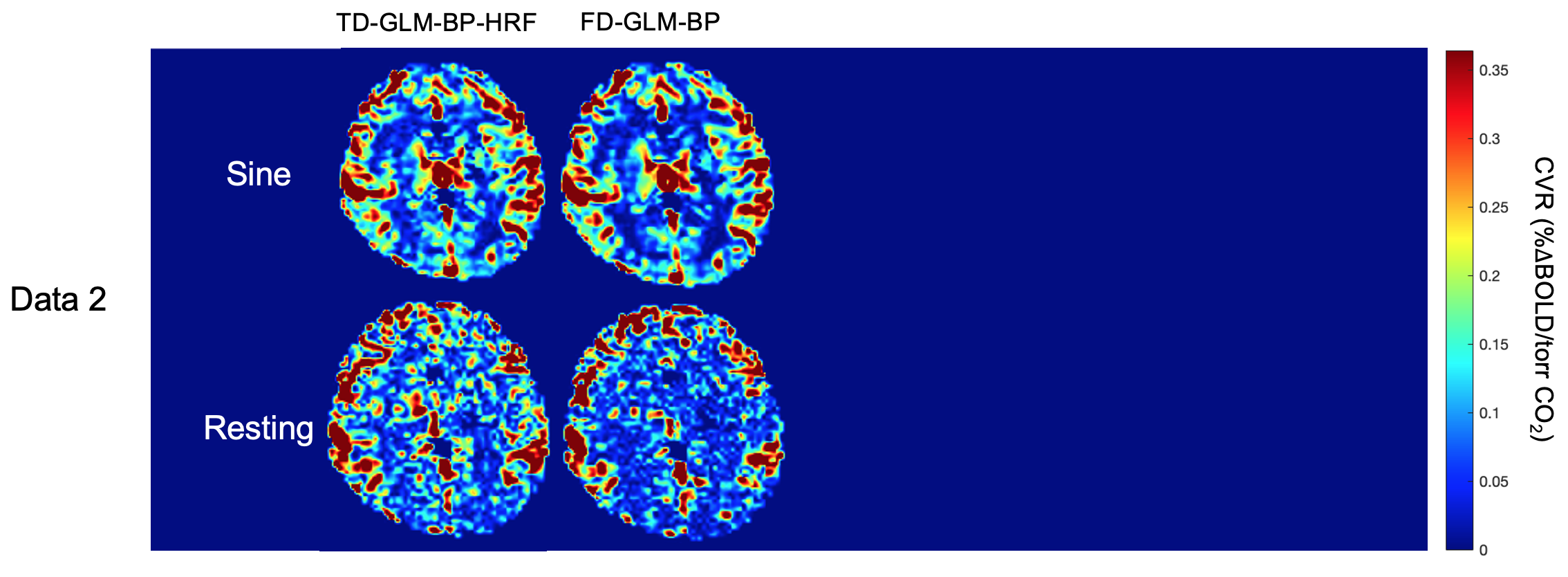

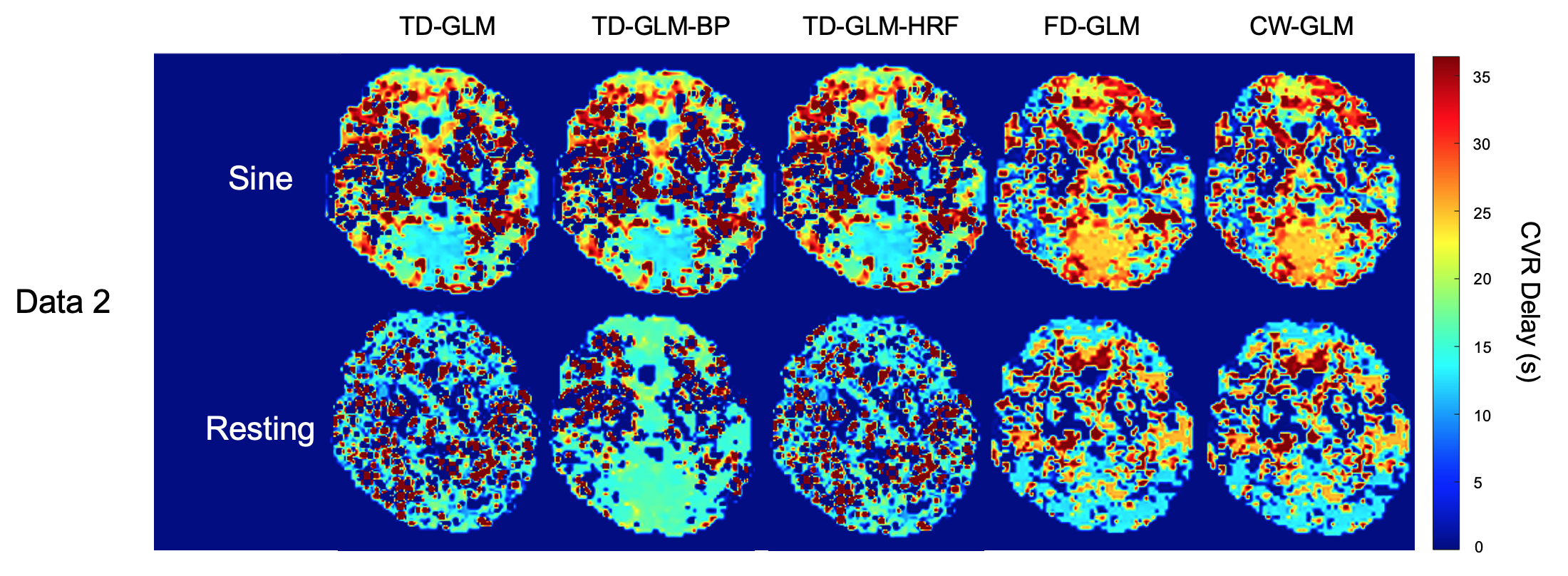

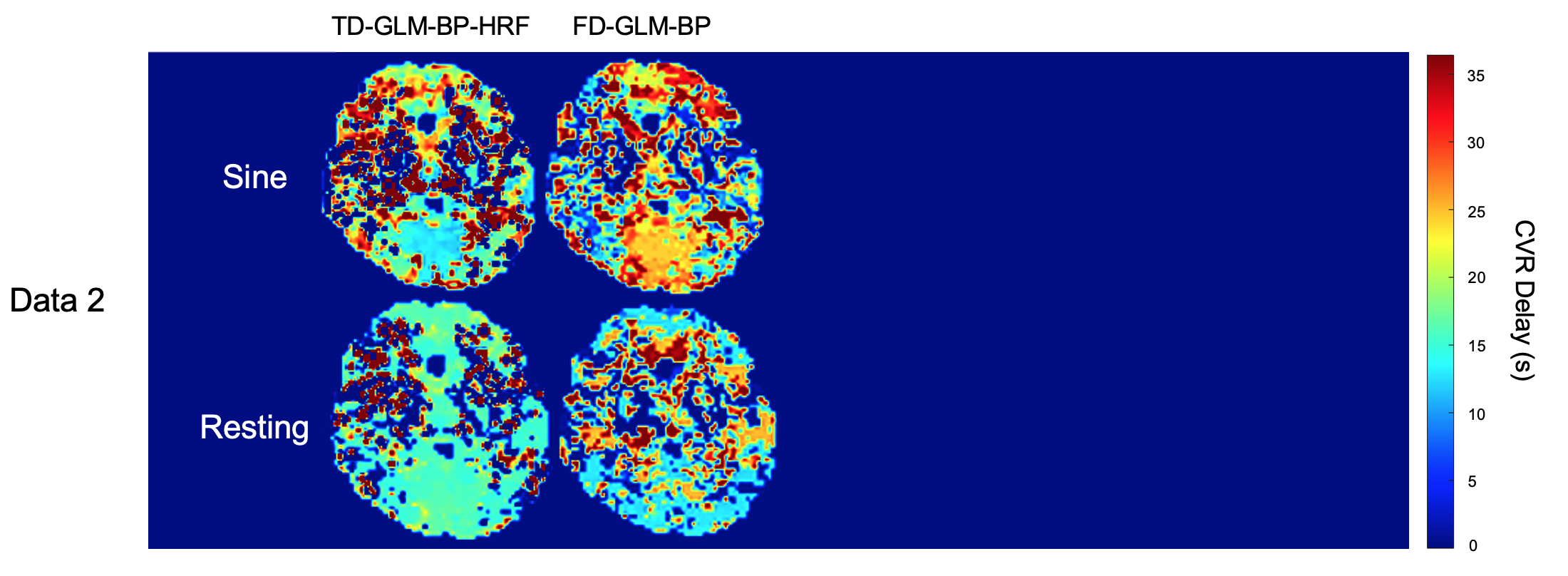


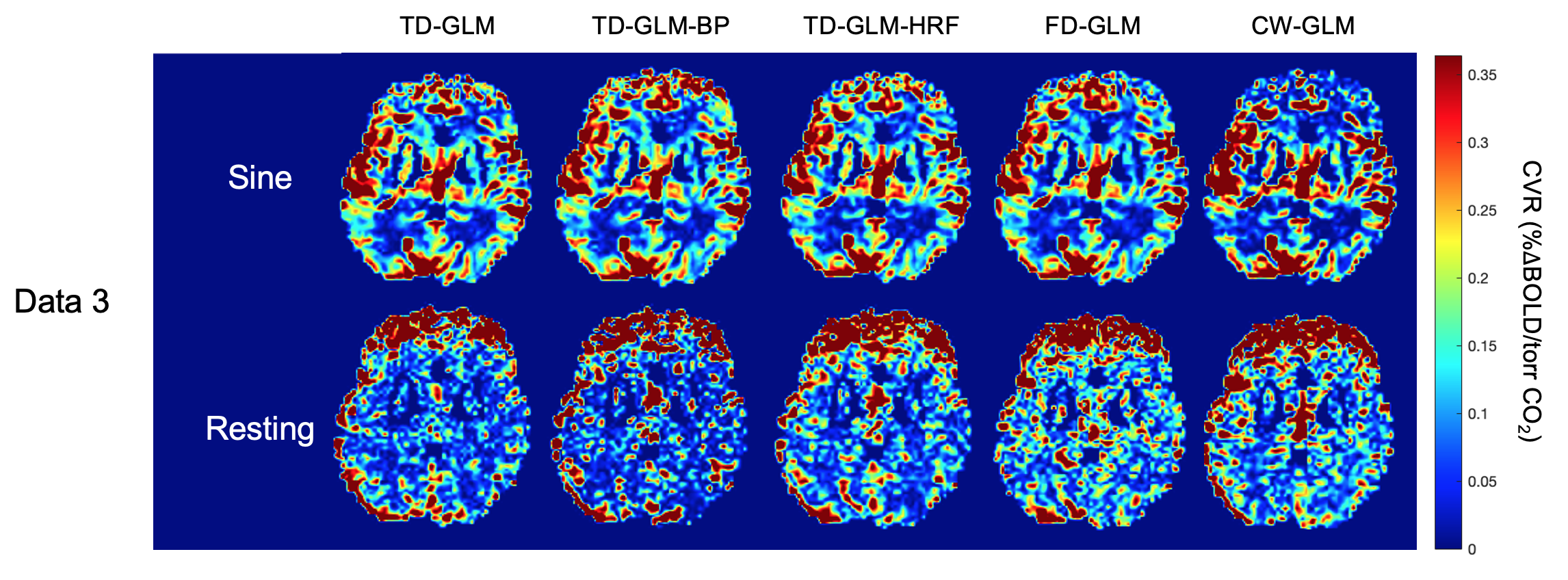

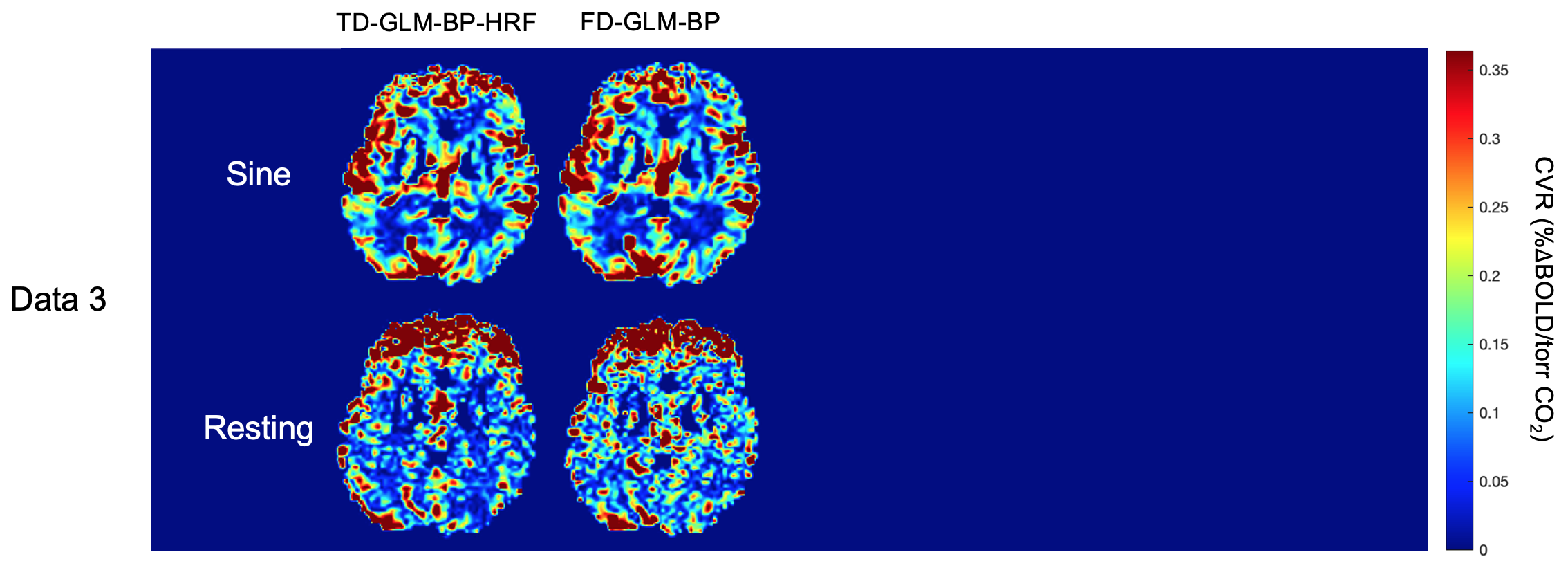

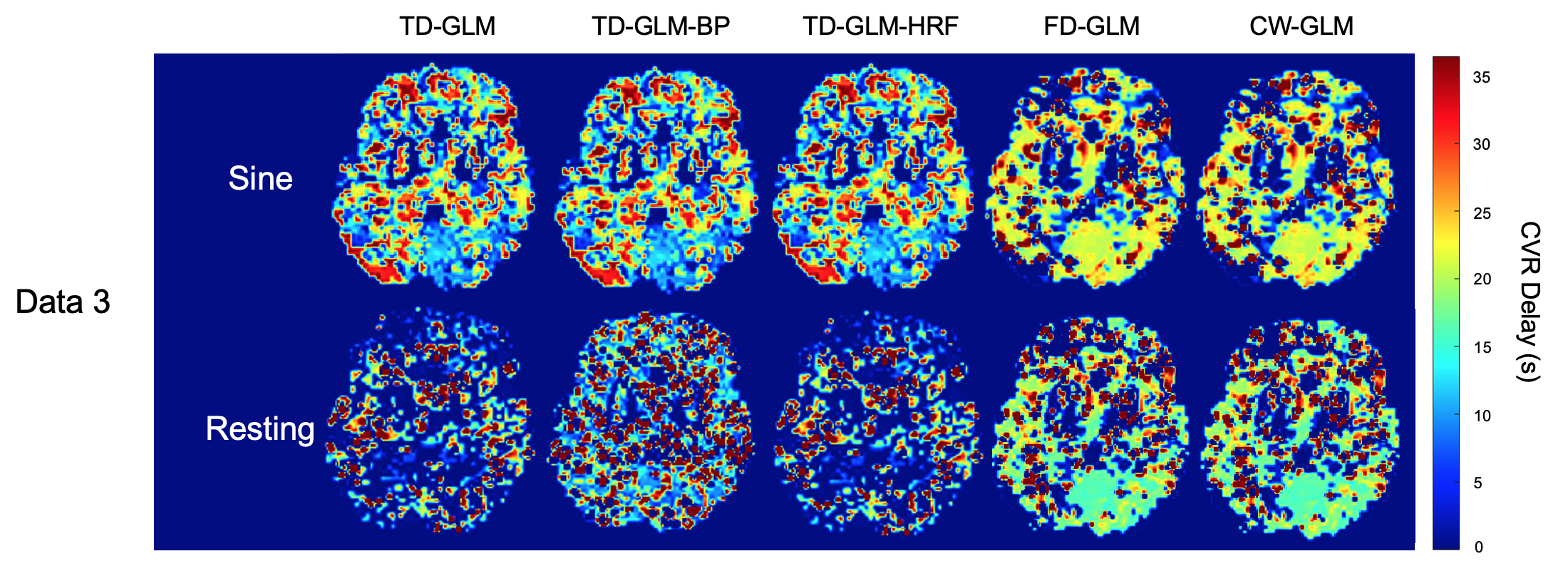

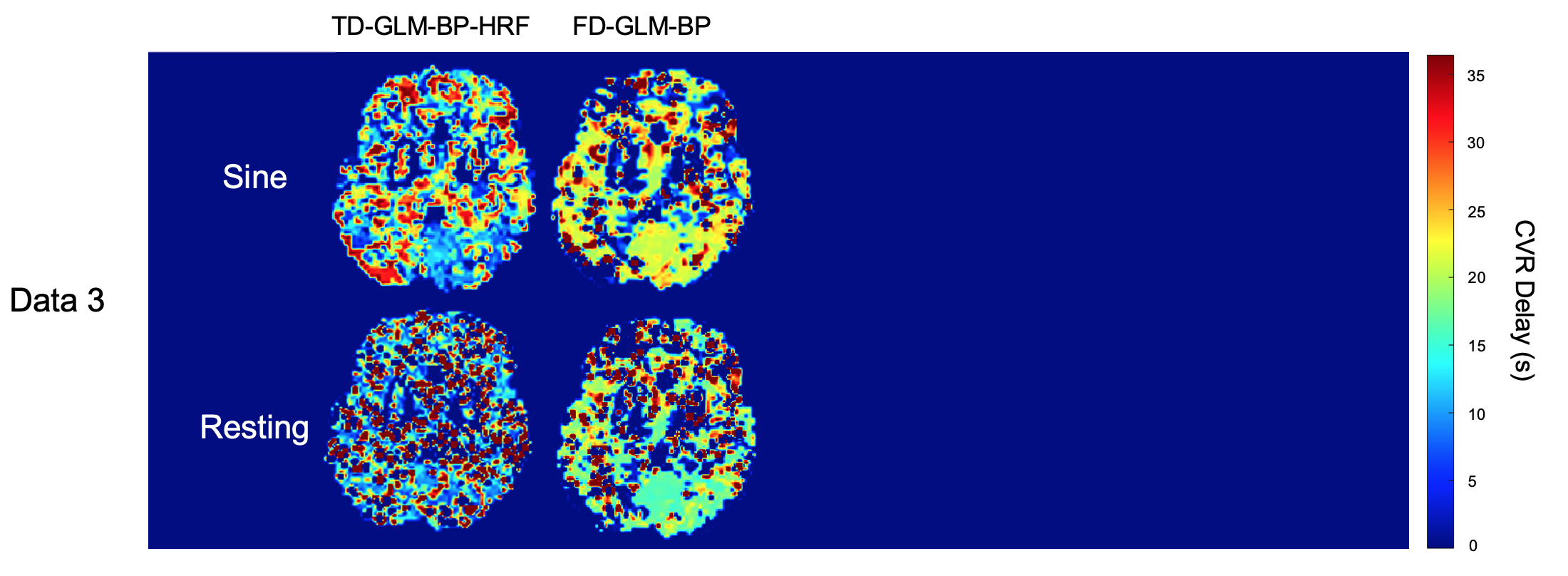


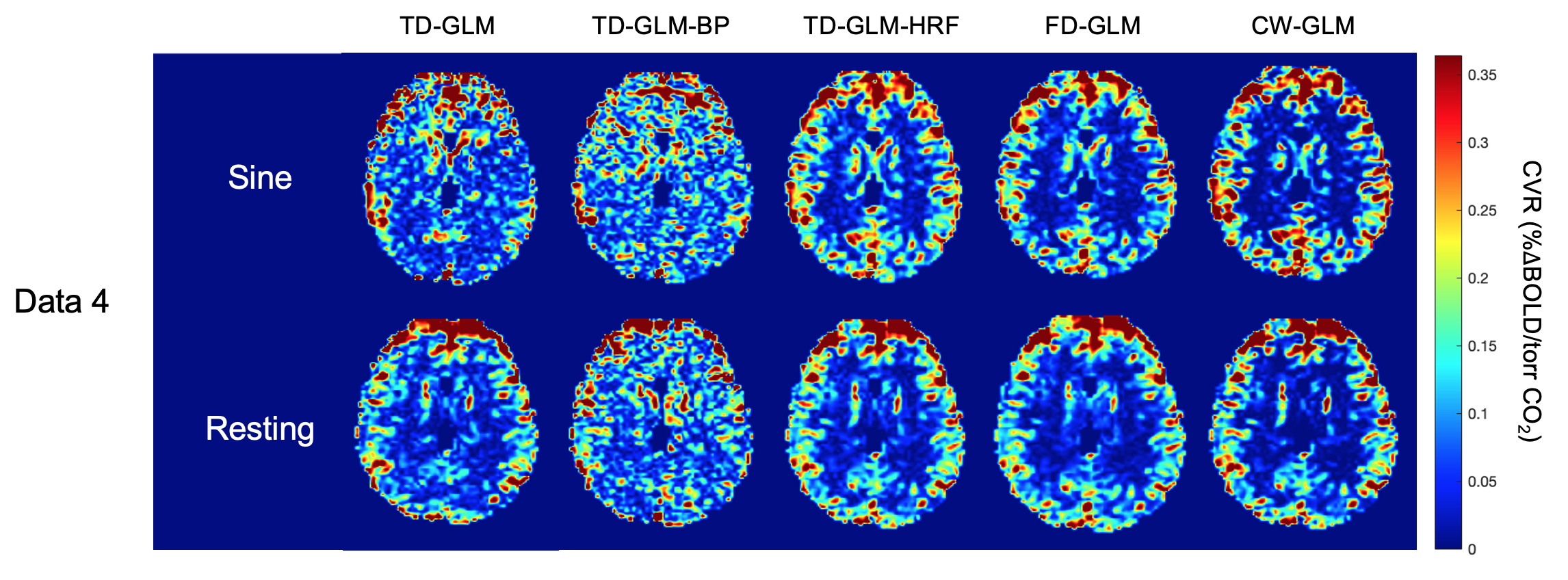

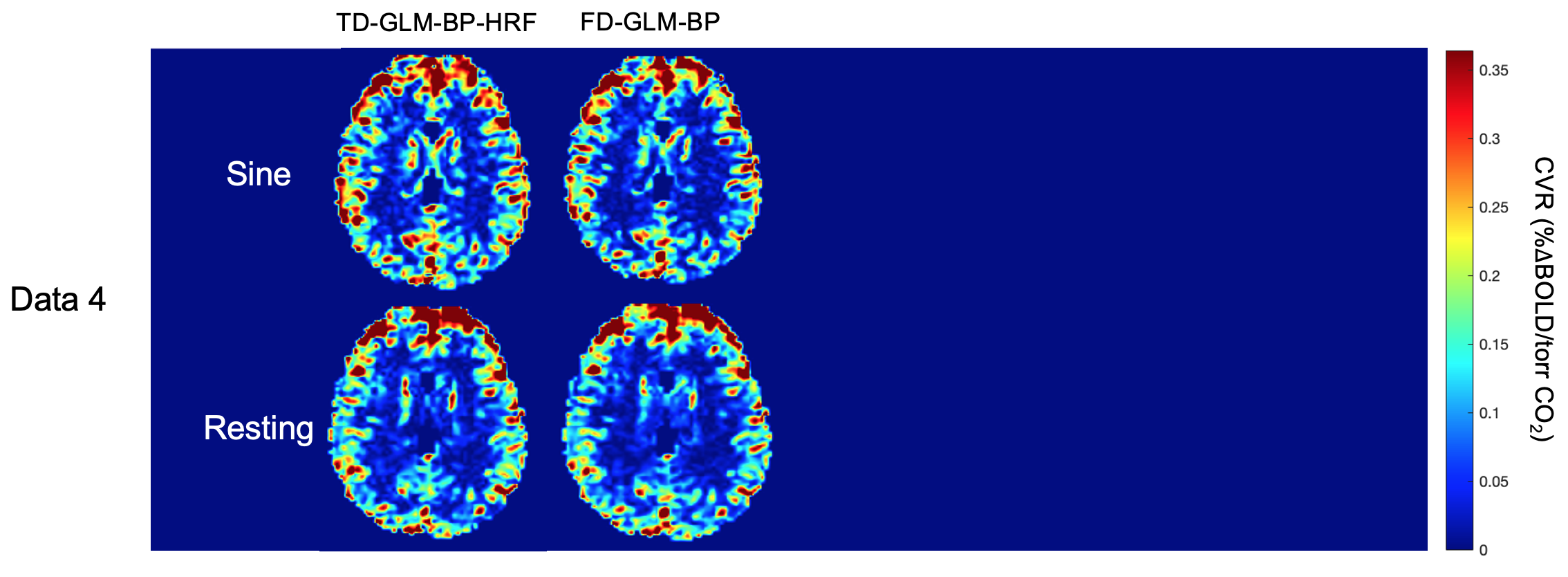

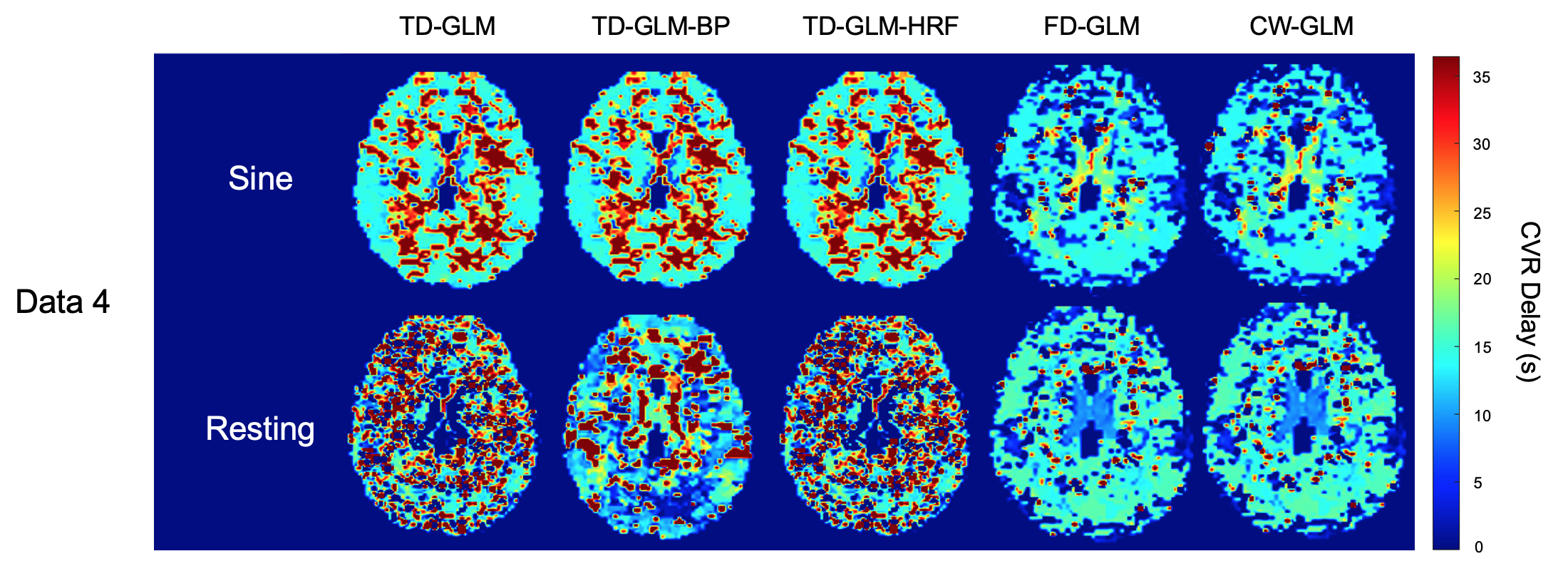

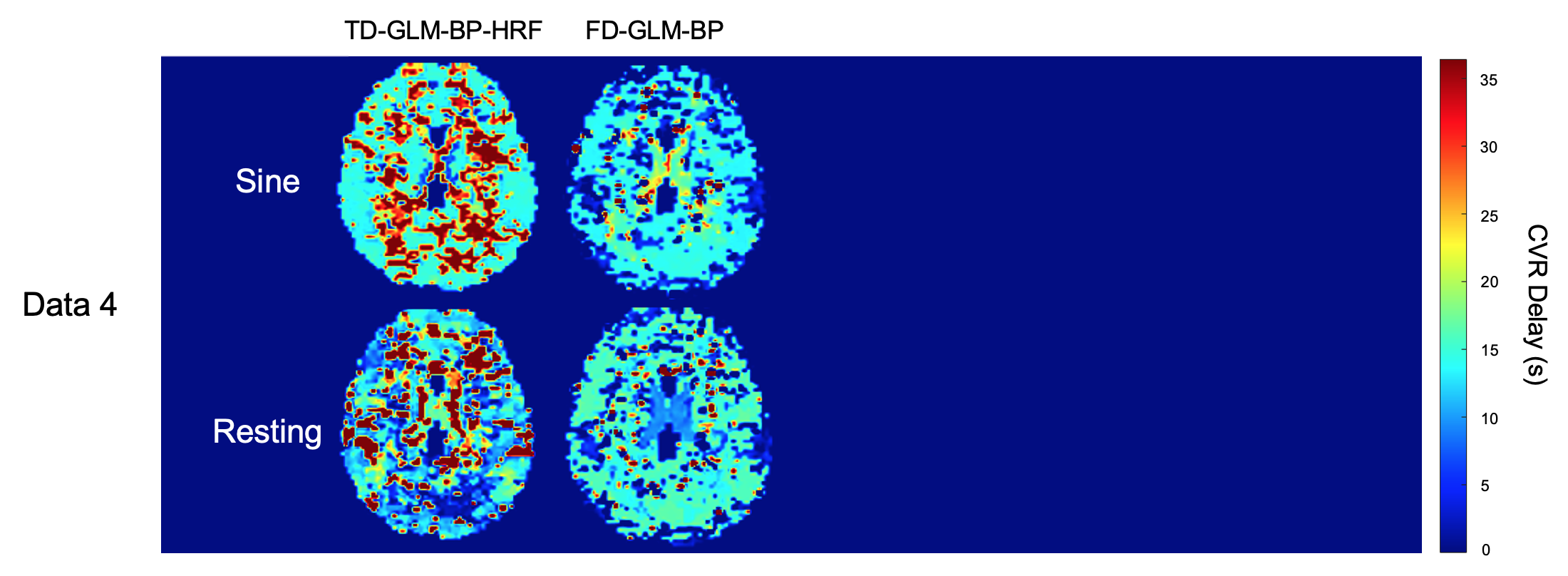


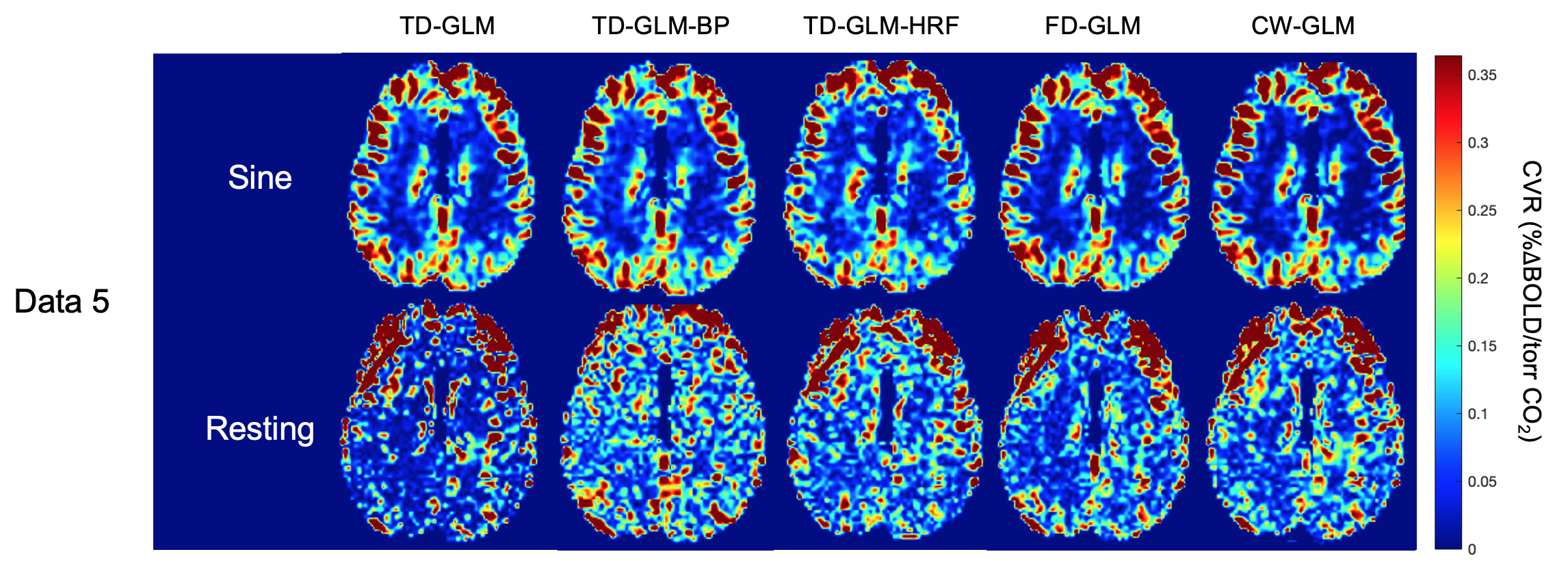

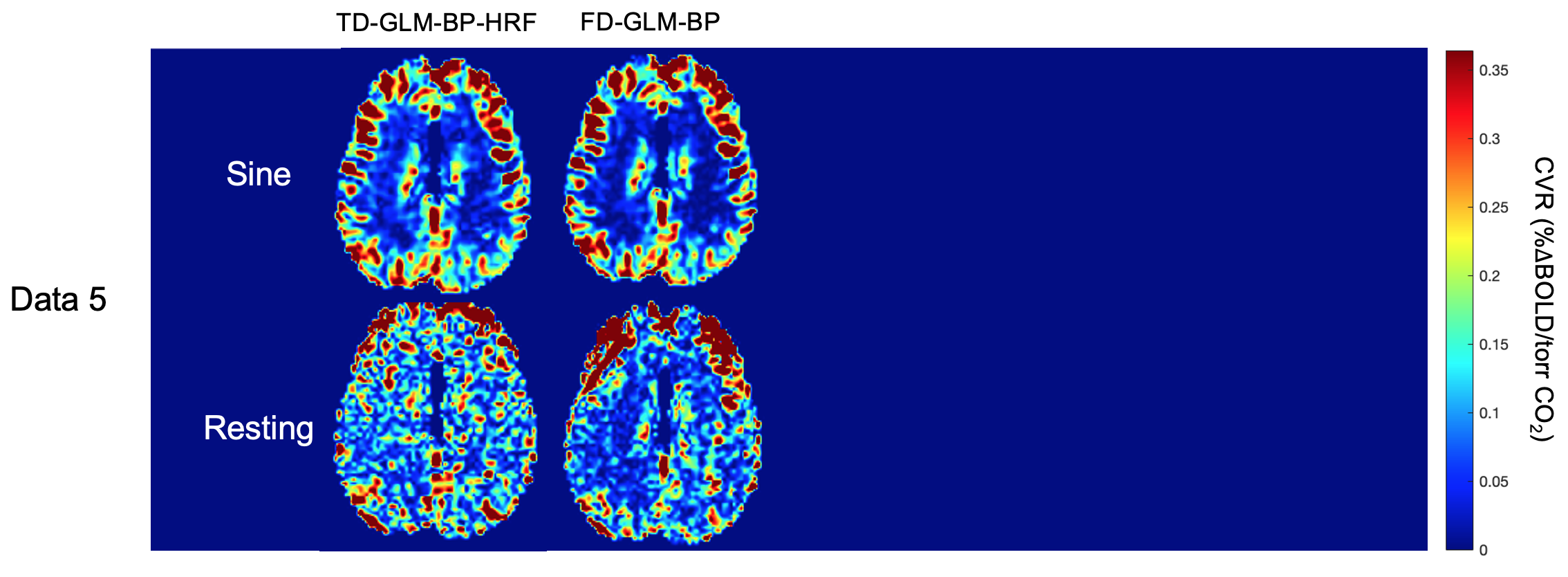

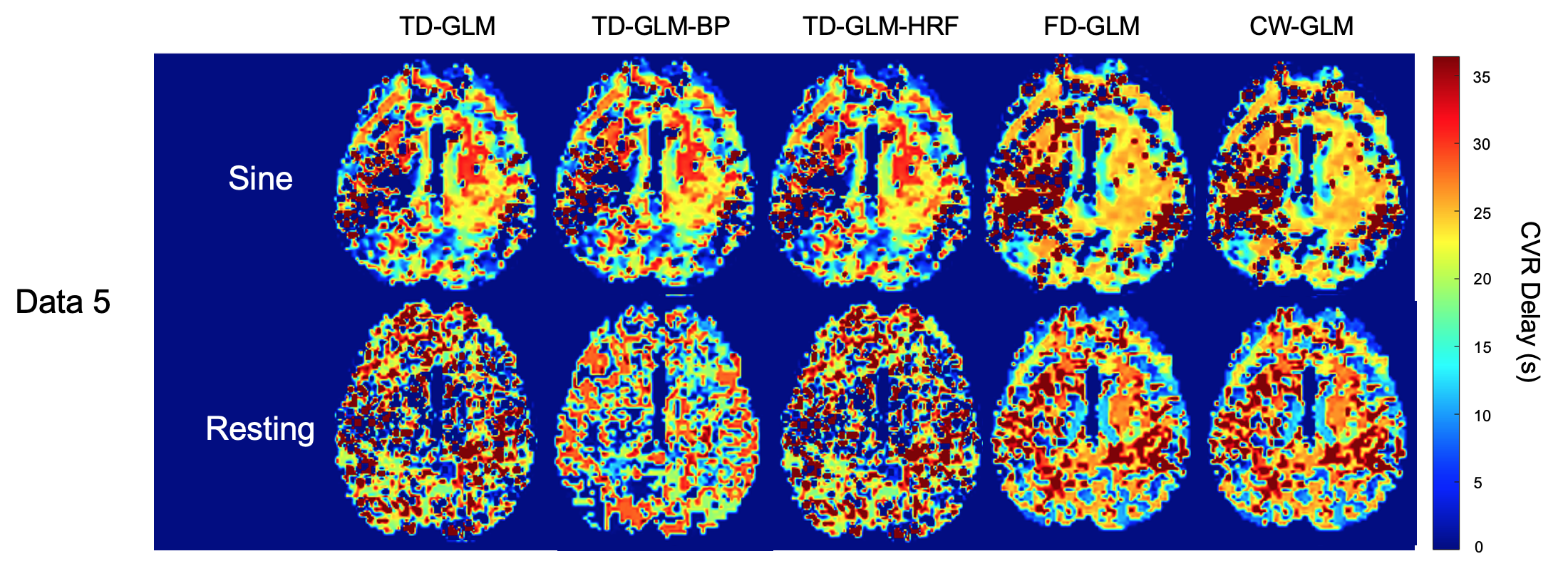

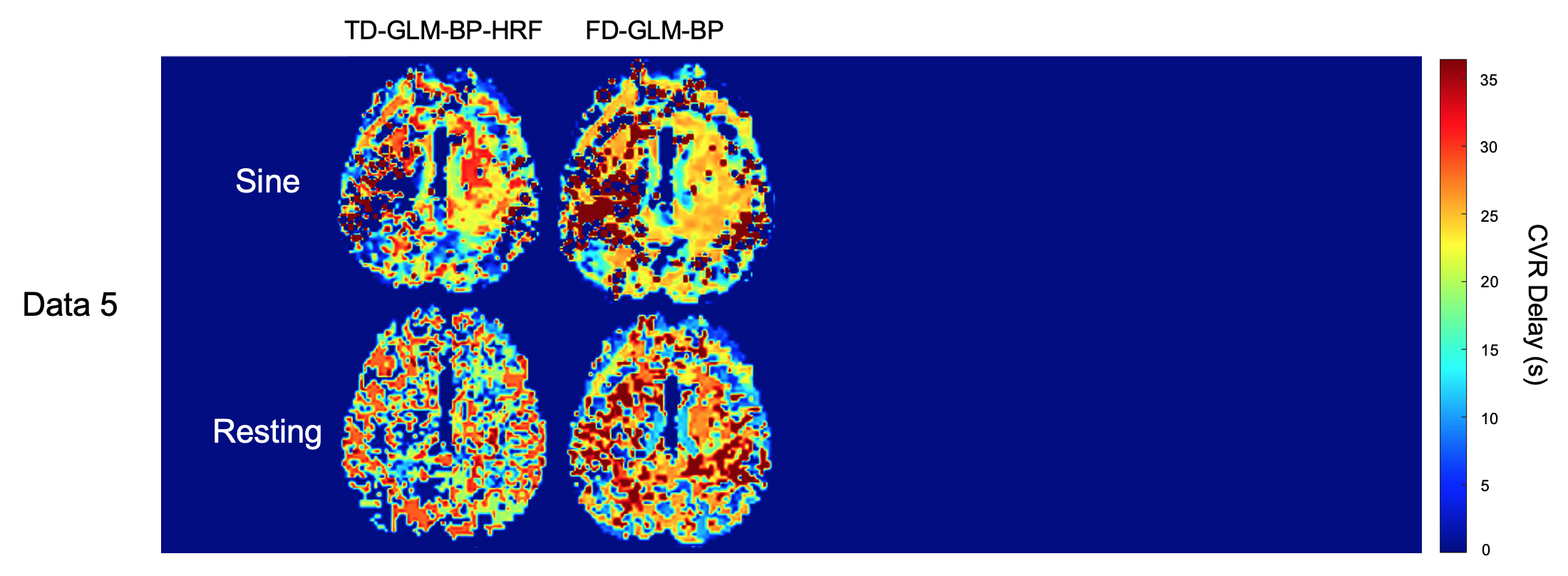


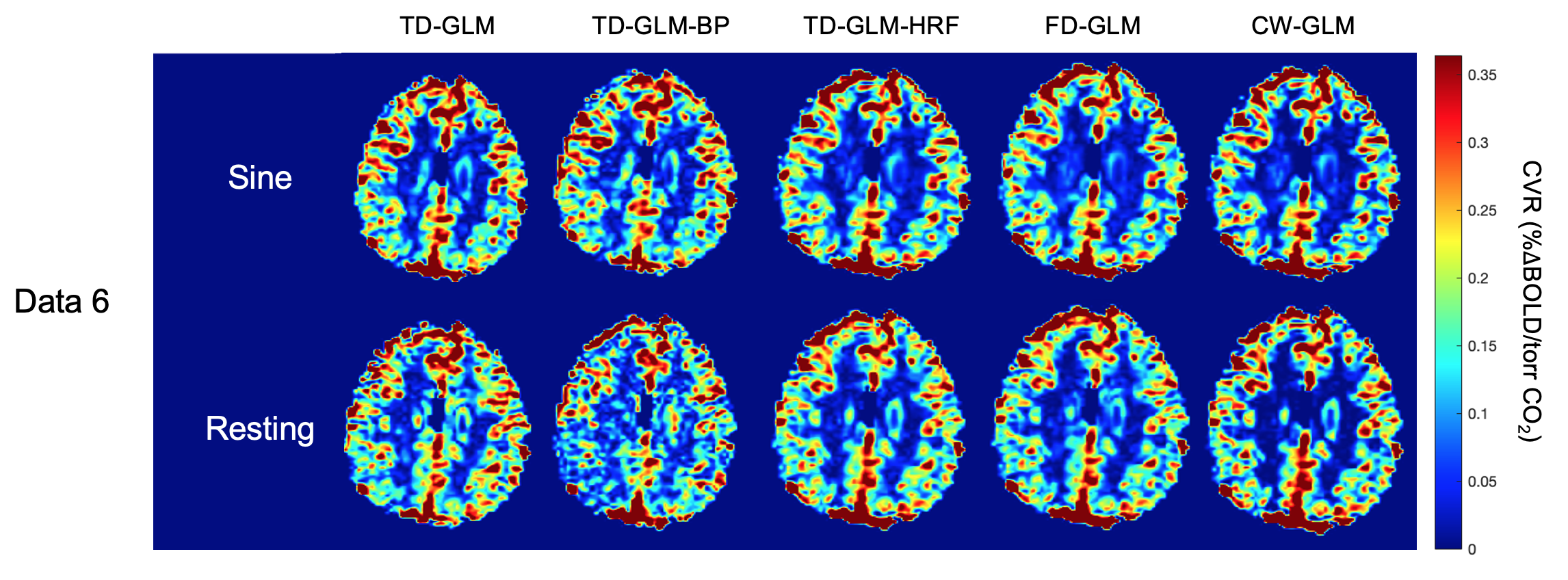

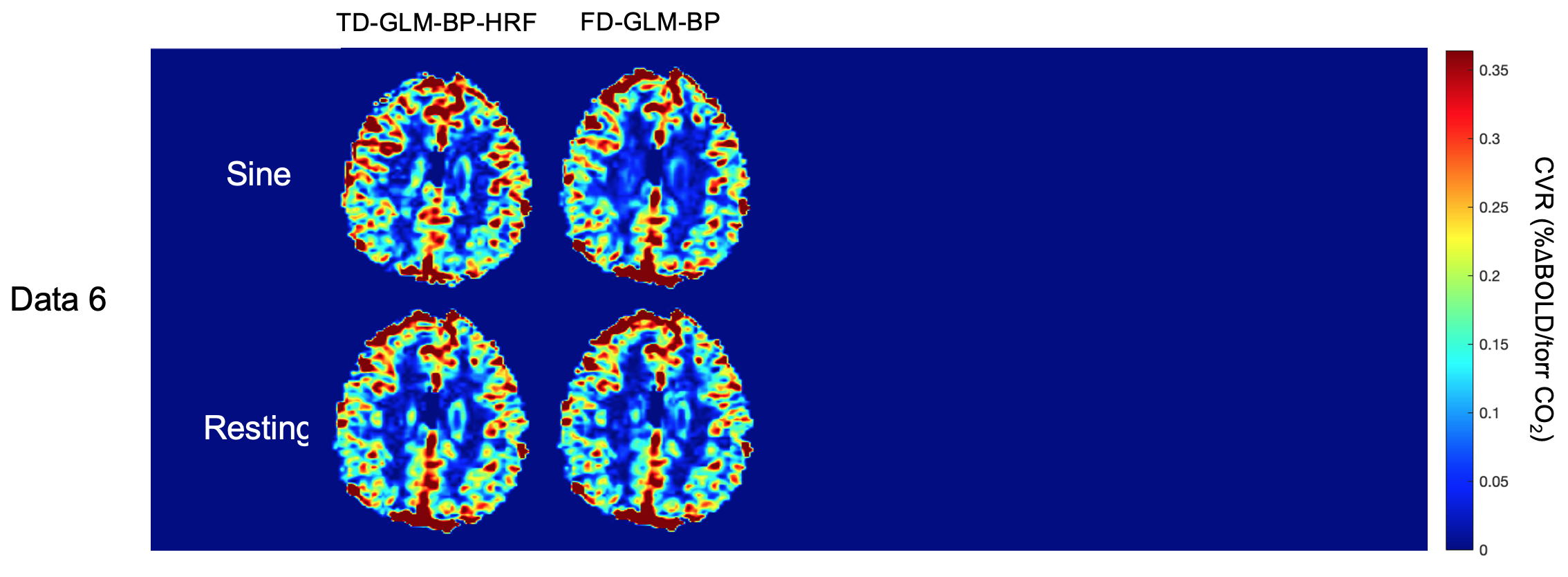

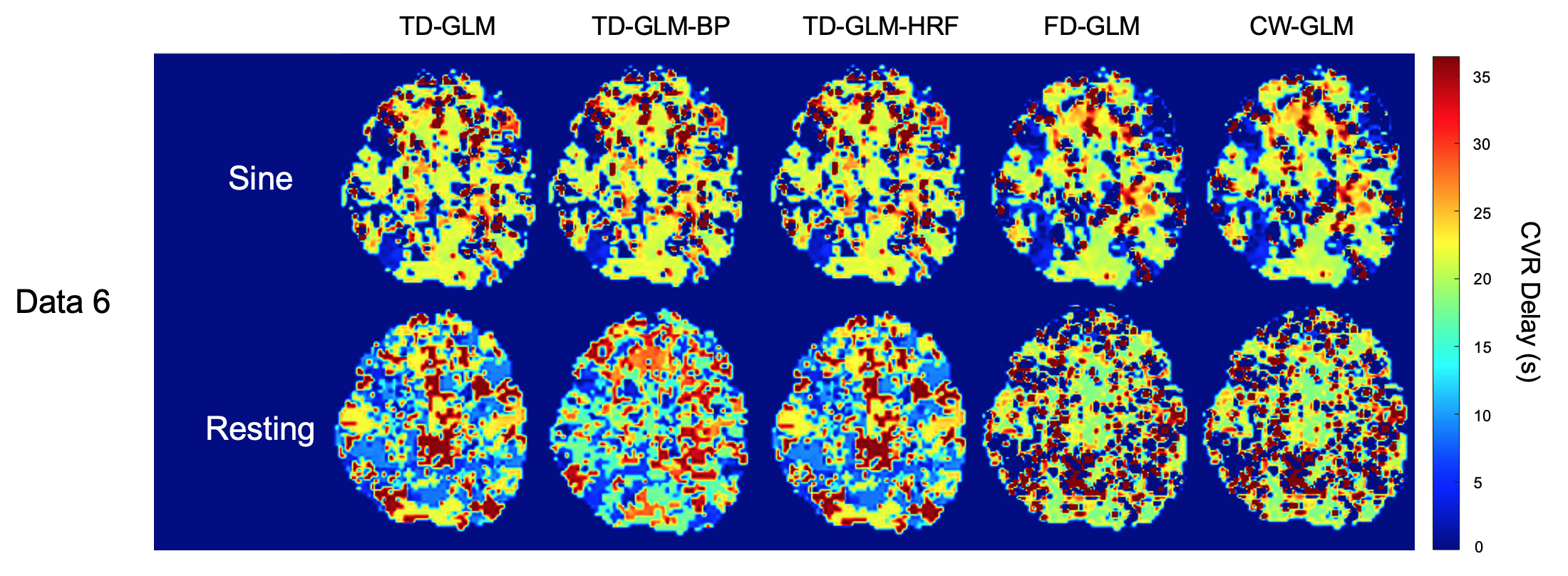

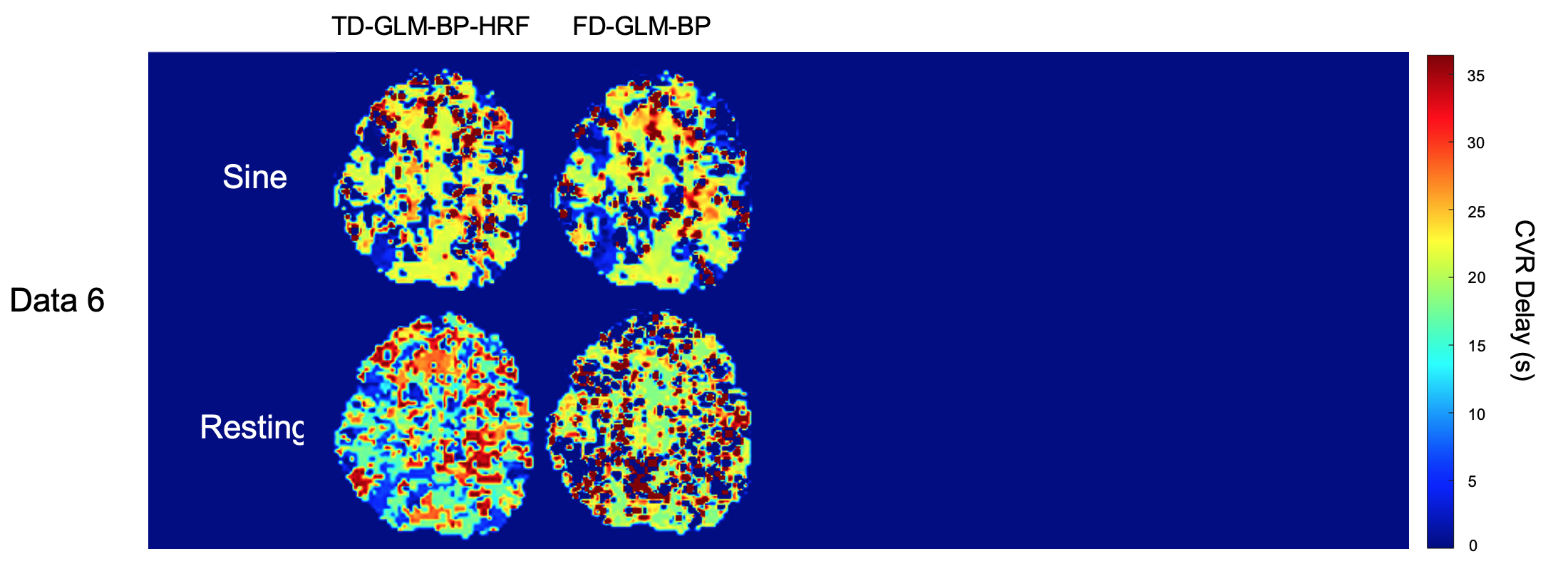


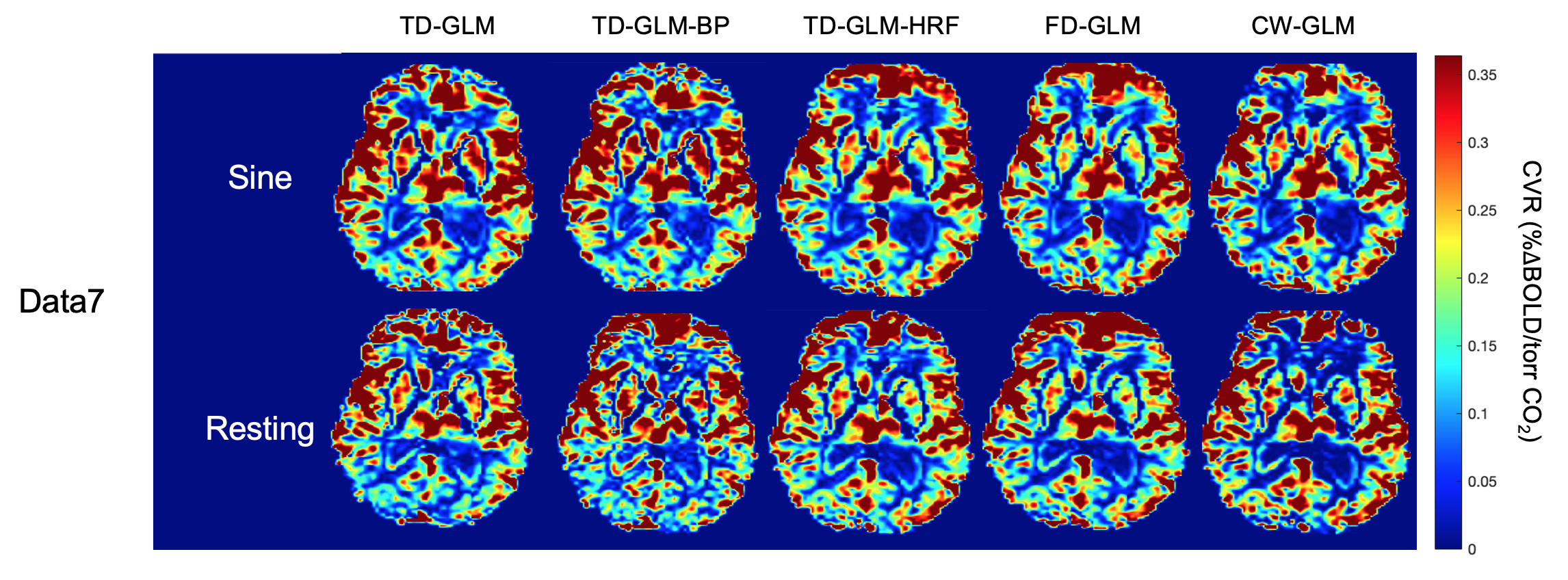

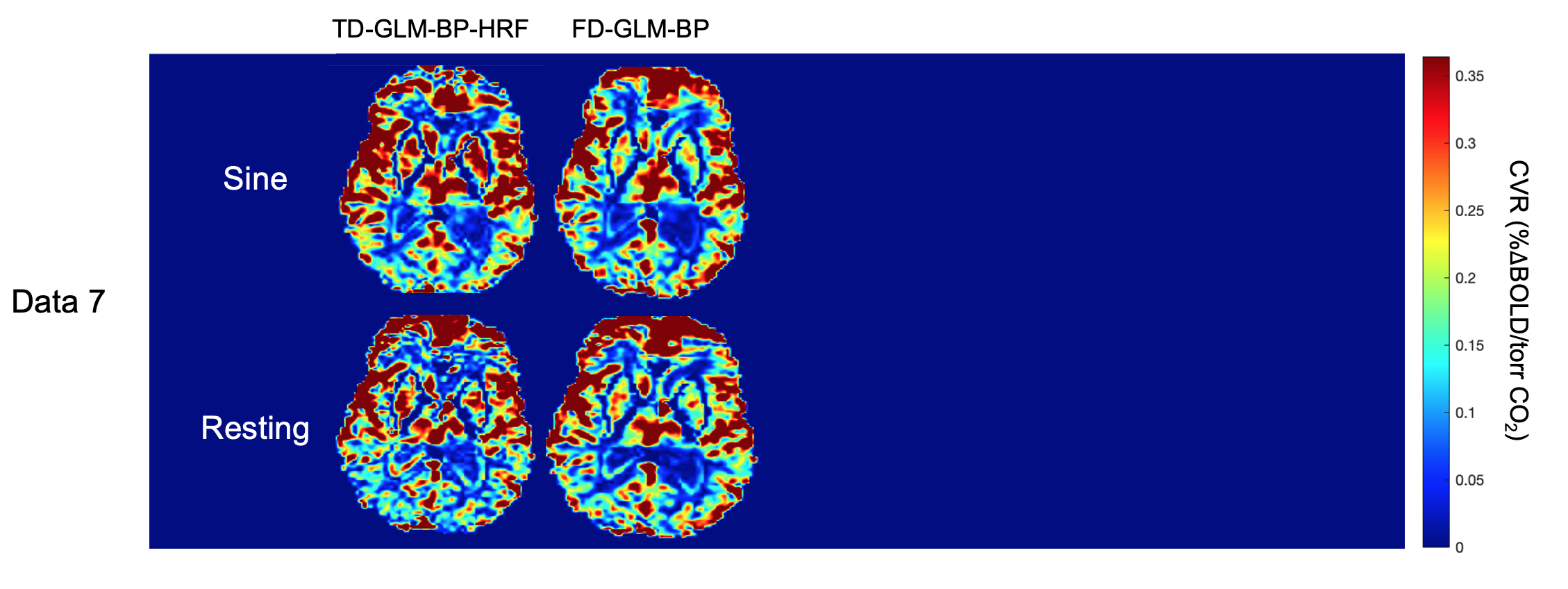

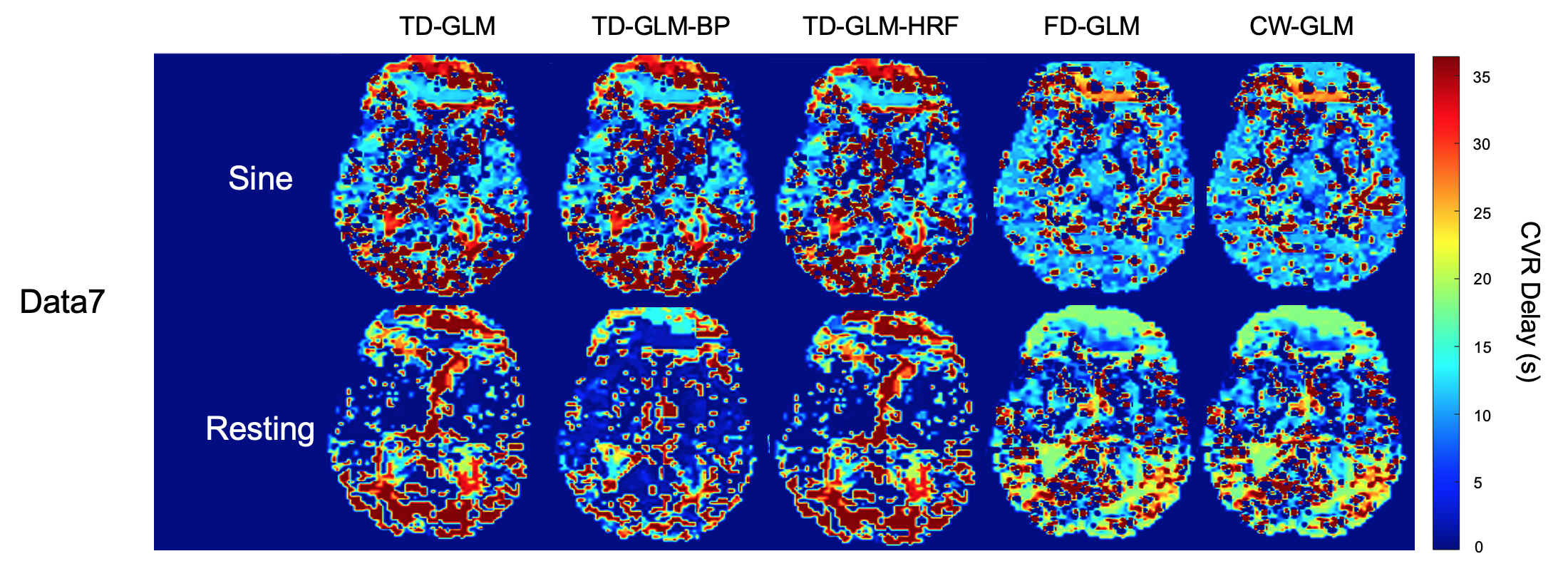

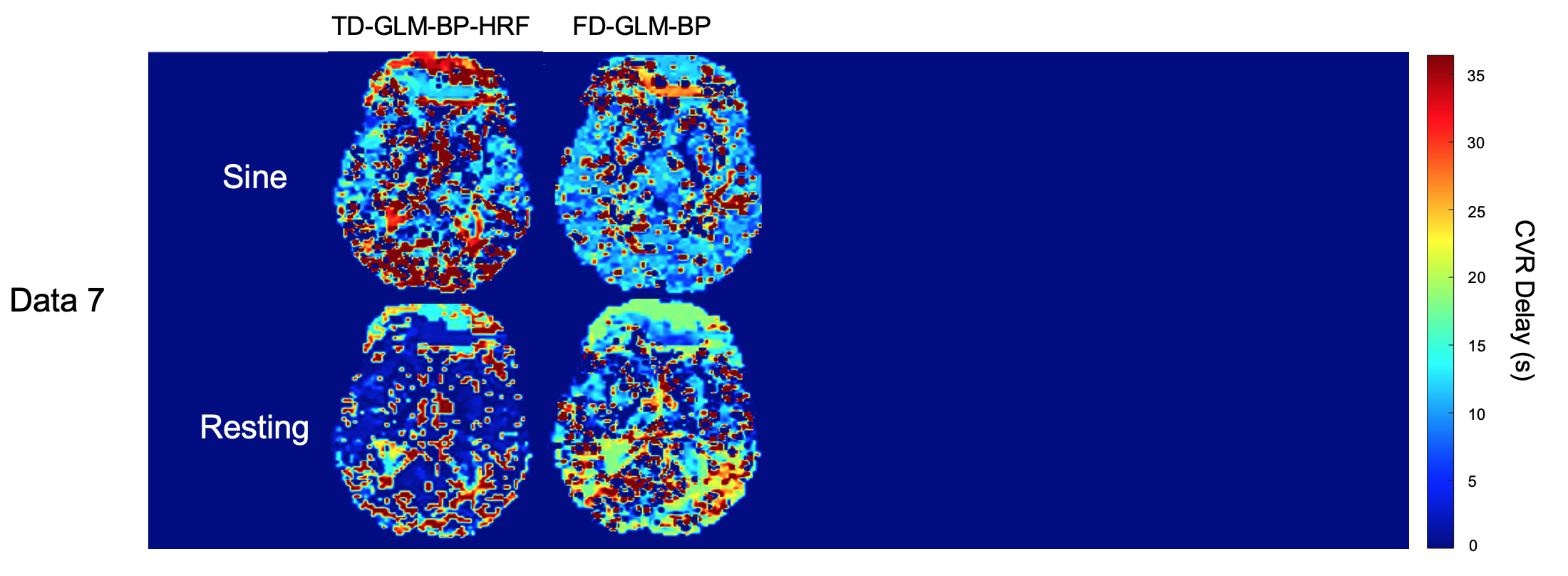


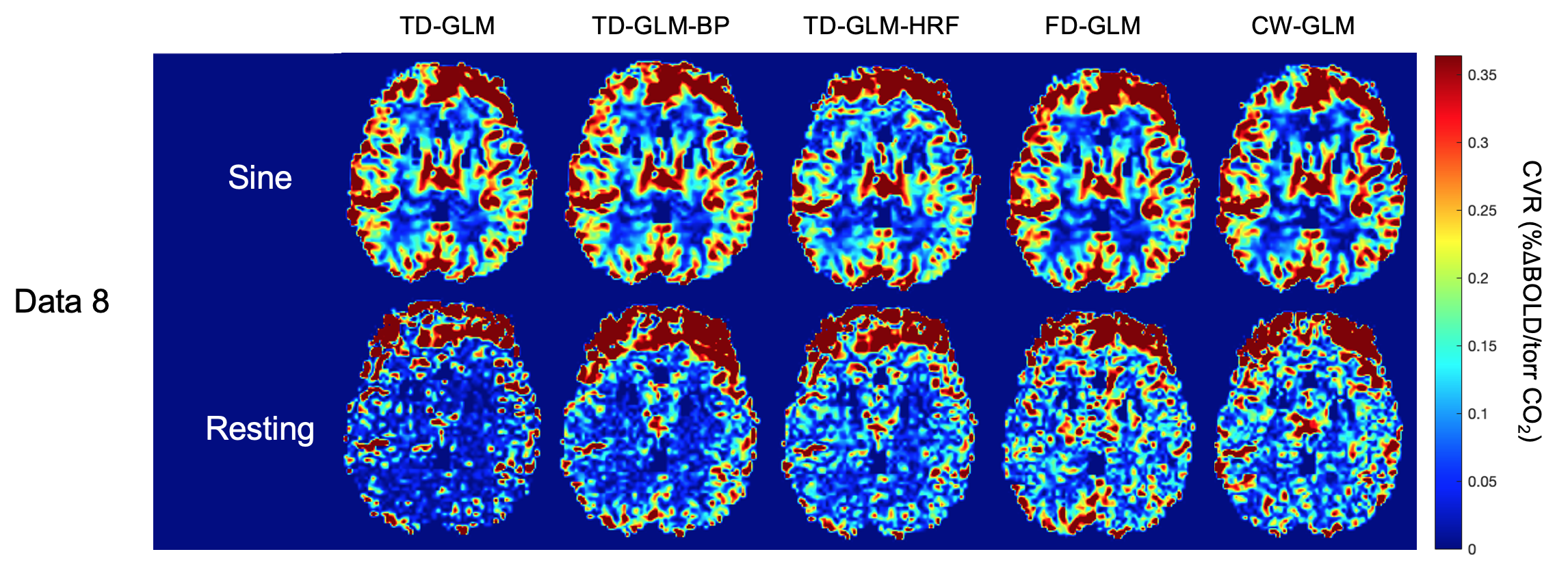

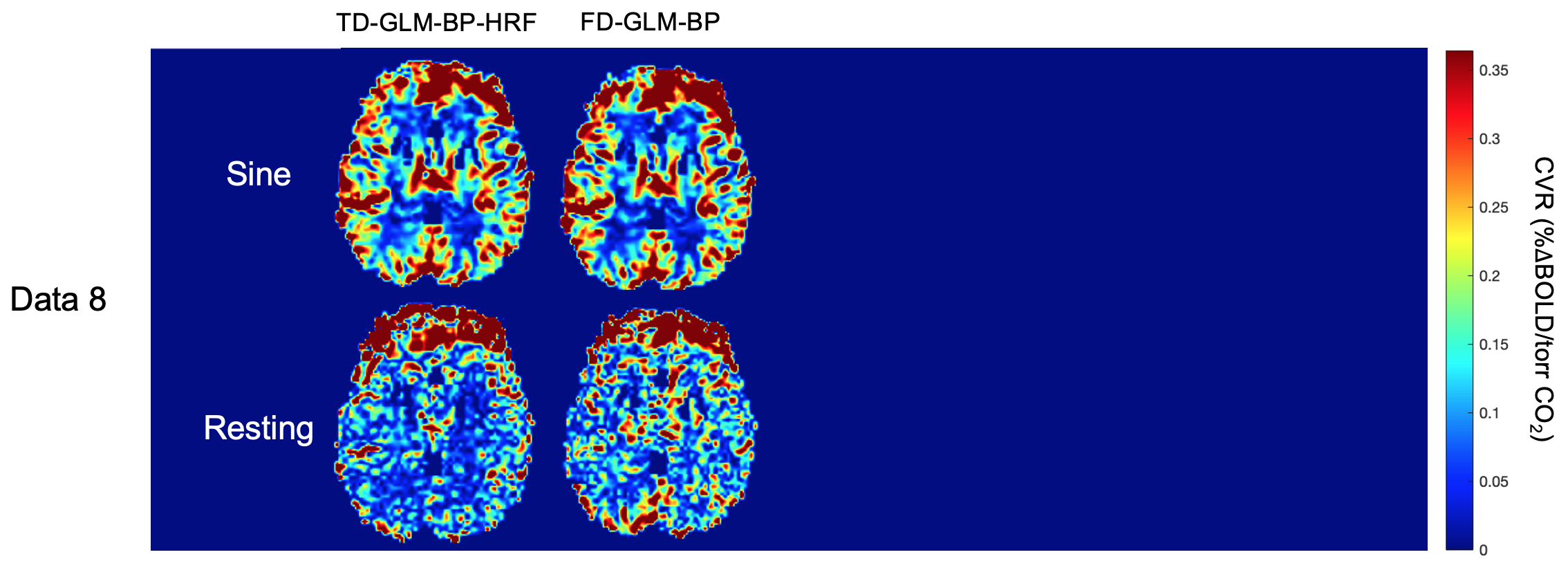

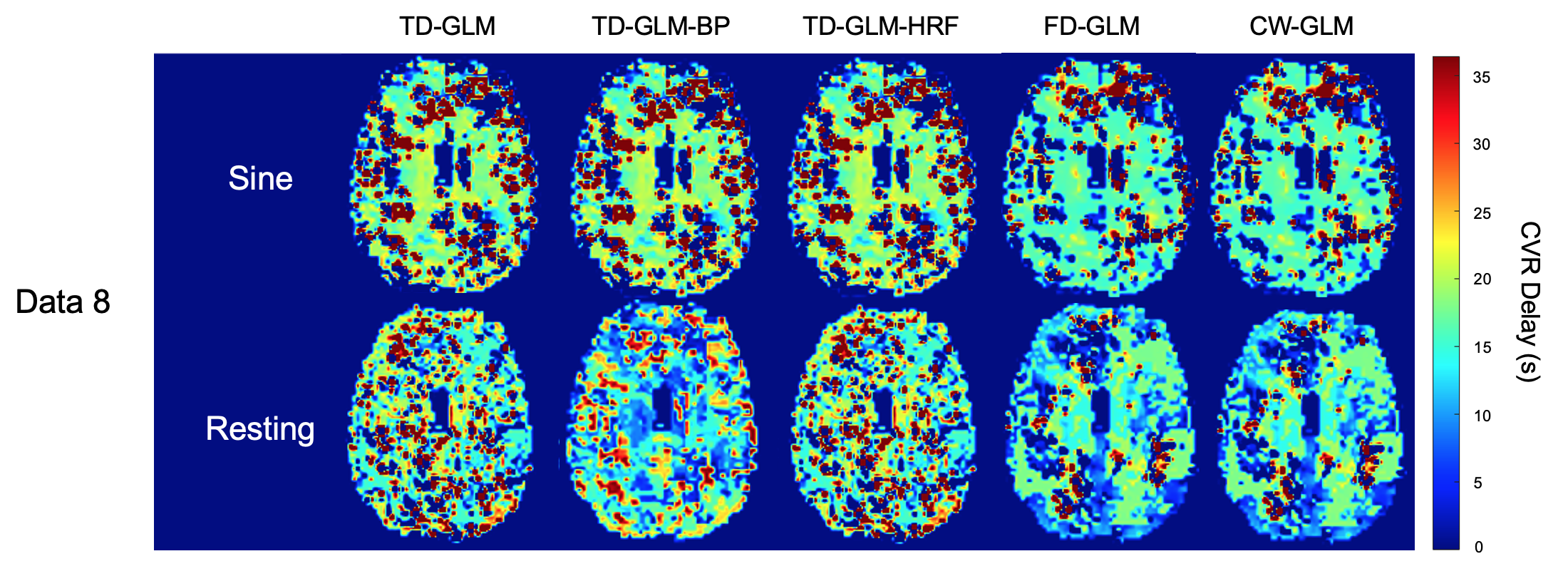

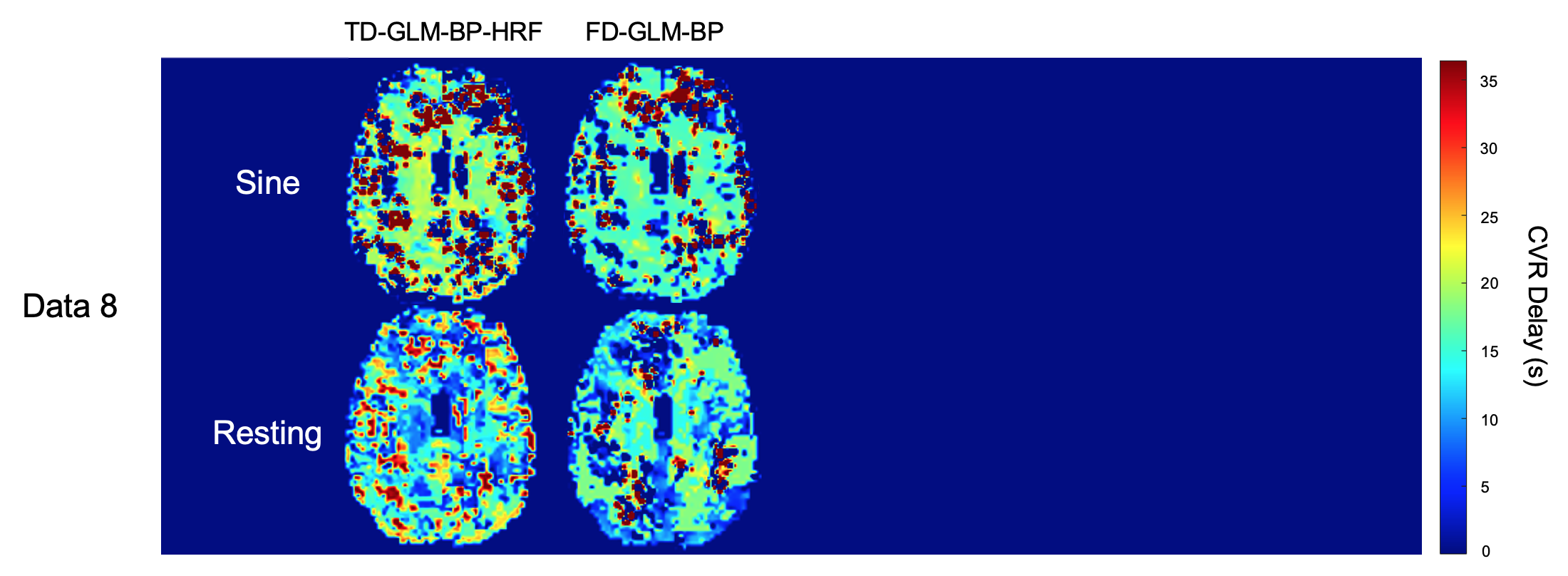


| **Method** | **Resting** | | **Sine** | |
| --- | --- | --- | --- | --- |
|  | **R square (GM)** | **R square (WM)** | **R square (GM)** | **R square (WM)** |
| **ALFF** | 0.66 $\pm$ 0.12 | 0.35 $\pm$ 0.12 | 0.74 $\pm$ 0.10 | 0.49 $\pm$ 0.13 |
| **fALFF** | 0.71 $\pm$ 0.11 | 0.41 $\pm$ 0.10 | 0.79 $\pm$ 0.09 | 0.57 $\pm$ 0.11 |

**Supplemental Table 1.** The regional voxel-wise correlation analysis between ALFF-based measurement and CVR from 36 subjects. (GM: grey matter; WM: white matter.)
